# Structural Insights into β-arrestin/CB1 Receptor Interaction: NMR and CD Studies on Model Peptides

**DOI:** 10.3390/ijms21218111

**Published:** 2020-10-30

**Authors:** Paula Morales, Marta Bruix, M. Angeles Jiménez

**Affiliations:** 1 Departamento de Química Física Biológica, Instituto de Química Física Rocasolano (IQFR-CSIC), Serrano 119, 28006 Madrid, Spain; marta.bruix@gmail.com; 2 Instituto de Química Médica (IQM-CSIC), Juan de la Cierva 3, 28006 Madrid, Spain

**Keywords:** CB1, β-arrestin1, NMR, Circular dichroism, GPCR

## Abstract

Activation of the cannabinoid CB1 receptor induces different cellular signaling cascades through coupling to different effector proteins (G-proteins and β-arrestins), triggering numerous therapeutic effects. Conformational changes and rearrangements at the intracellular domain of this GPCR receptor that accompany ligand binding dictate the signaling pathways. The GPCR-binding interface for G proteins has been extensively studied, whereas β-arrestin/GPCR complexes are still poorly understood. To gain knowledge in this direction, we designed peptides that mimic the motifs involved in the putative interacting region: β-arrestin1 finger loop and the transmembrane helix 7-helix 8 (TMH7-H8) elbow located at the intracellular side of the CB1 receptor. According to circular dichroism and NMR data, these peptides form a native-like, helical conformation and interact with each other in aqueous solution, in the presence of trifluoroethanol, and using zwitterionic detergent micelles as membrane mimics. These results increase our understanding of the binding mode of β-arrestin and CB1 receptor and validate minimalist approaches to structurally comprehend complex protein systems.

## 1. Introduction

The therapeutic effects of cannabinoids have long been known; however, it was not until a few decades ago that their mechanism of action was elucidated. In the late 1980s, receptors targeted by phytocannabinoids were identified in rat brain [1]. Subsequent cloning of this G protein-coupled receptor (GPCR) consolidated the discovery of the first cannabinoid receptor, CB1 [2]. 

CB1 is highly expressed throughout the central nervous system, being one of the most abundant GPCRs in the human brain [3]. CB1 receptors are also found in the peripheral nervous system, as well as in other organs and tissues including endocrine glands, spleen, heart or the gastrointestinal tract. This expression pattern confers upon CB1 a relevant role in the modulation of numerous physiopathological processes including memory processing, pain regulation or neurodegeneration [3,4,5,6]. A growing body of research supports the notion that CB1 represents a promising target for the development of novel drugs for the treatment of diverse pathologies including neurodegenerative, cancer or metabolic disorders [7,8,9,10,11,12,13,14,15].

The activation of this receptor involves complex signaling pathways whose mechanisms still need to be fully unraveled. CB1 receptors are mainly coupled to G_αi/o_ proteins, negatively to adenylyl cyclase (AC) and positively to mitogen-activated protein kinases (MAPK). Other G_α_ isoforms, such as G_αs_ [16,17,18,19,20], and G_αq/11_ [21], have also been shown to couple to CB1 under particular circumstances. Upon CB1-mediated G_αi/o_ coupling, AC is inhibited, thus inhibiting the conversion of ATP to cyclic AMP (cAMP). CB1 activation also triggers increased phosphorylation of extracellular signal-regulated kinase 1/2 (pERK1/2). The G_βγ_ subunits, dissociated from the G_αi/o_, activate G protein-coupled inwardly rectifying potassium channels (GIRKs) and phosphatidylinositide-3-kinase (PI3K) and inhibit voltage-gated calcium channels (VGCC) [22,23]. Moreover, upon G protein activation, G protein-coupled receptor kinases (GRKs) phosphorylate specific serines and threonines in the intracellular domain of the receptor, inducing β-arrestin recruitment [24]. Two β-arrestin isoforms have been shown to be recruited to CB1: β-arrestin1, which triggers the activation of the ERK pathway, and β-arrestin2, which leads to receptor desensitization and internalization [25,26,27]. 

In addition to the G-protein canonical pathway, G-protein independent β-arrestin recruitment to GPCRs was recently demonstrated in diverse GPCRs [28,29,30,31]. Most known CB1 agonists activate the receptor through both G protein and β-arrestin signaling pathways in an unbiased manner [32,33,34]. The search of biased ligands has greatly increased in the last years in the cannabinoid field [27,35,36]. These functionally selective ligands should be able to stabilize distinct GPCR states that vary the ability of the receptor to activate specific transducers, such as activation of different G-proteins and/or signaling through β-arrestins, leading to different physiologic outcomes [37,38,39,40]. This may provide optimized therapeutic results while avoiding undesired effects mediated through specific pathways. However, the design of biased ligands remains a challenge, since the precise nature of conformational receptor changes inducing pathway specificity has not yet been unraveled. 

From a structural perspective, the G-protein activation mechanism of GPCRs has been extensively studied [41,42,43,44,45]. However, GPCR conformational changes that result in β-arrestin coupling have not yet been fully explained. High-resolution crystal structures of the CB1 receptor, in its G-protein inactive and active states, have been recently reported [46,47,48]. Moreover, cryoelectron microscopy structures of CB1-G_i_ complexes bound to potent agonists were recently resolved, providing insights into the G-protein coupling activation mechanism of CB1 [49,50]. Nevertheless, no CB1 receptor structure in complex with β-arrestin has been determined to date.

In an effort to better understand the β-arrestin1/CB1 receptor interface, we designed peptides that mimic the motifs potentially involved in the interacting region. Circular dichroism (CD) and nuclear magnetic resonance (NMR) studies were performed to help to determine the conformation of these peptides and characterizing the key structural features of their interaction. 

## 2. Results and Discussion

### 2.1. Peptide Design

**Figure 1:**
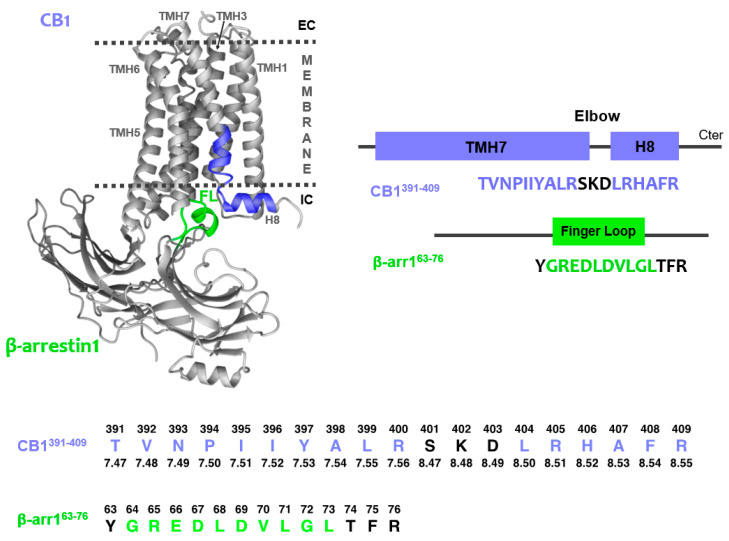
CB1/β-arrestin1 complex model [templates: PDB 5XRA (CB1 receptor active state crystal structure) and 5W0P (Rho/β-arrestin complex)]. The CB1/β-arrestin1 interface to be studied is colored (green: β-arr1^63-76^; blue: CB1^391-409^) while other domains in the GPCR and the scaffolding protein are represented in grey. Note that β-arrestin finger loop (FL) is not helical in other reported GPCR/β-arrestin complexes [51,52,53]. At the bottom, the absolute sequence numbers are shown above the peptide sequences; in the case of CB1^391-409^ the Ballesteros and Weinstein numbering of GPCRs is indicated below the sequence.

Since no experimental structure of the CB1/β-arrestin complex has been reported to date, the design of peptides that mimic the interacting motifs in the CB1/β-arrestin1 complex was based on previously reported data on GPCR/arrestin interface. To illustrate the region of interest, we developed a model (Figure 1) using as templates the crystal structures of CB1 receptors in their active state (PDB 5XRA) and the rhodopsine/arrestin complex (PDB 5W0P), which was the only GPCR/arrestin complex available at the beginning of this work. In the three GPCR/ β-arrestin complexes reported this year, the interacting regions are analogous, although the β-arrestin finger loop displays structural diversity [51,52,53].

Concerning β-arrestin1, it has been reported that its finger loop region (FL, Figure 1) is a critical determinant of arrestin coupling to GPCRs [51,52,53,54,55,56]. The finger loop region was first identified by sequence alignment of several β-arrestins (Supplementary Table S1). Then, the potential effects of including the preceding and following residues on helical tendency and solubility was examined by the AGADIR and Protparam webservers [57,58]. The sequence for the β-arrestin1 model peptide was selected as the shortest sequence having the highest helical tendency and being the most soluble at the neutral (or slightly acidic) pH values used in the NMR study (note that peptide solubility is usually minimal at the isoelectric point, pI; Supplementary Table S1). This β-arrestin1 model peptide (β-arr1^63-76^) includes the preceding residue and three after the finger loop motif, as indicated in Figure 1. The β-arrestin finger loop is structurally diverse in the reported GPCR/β-arrestin complexes, adding interest to study the structure of this region by itself. 

As shown in this figure, the general topology of GPCRs encompasses seven transmembrane helices (TMH) connected by intracellular and extracellular loops and a short cytoplasmic helical domain (H8) extending from TMH7. This helical segment is oriented in parallel to the membrane surface and perpendicularly to the TMH bundle. 

The scarce structural knowledge available on GPCR/arrestin complexes indicates, as seen in the model of CB1/β-arrestin1 (Figure 1), that the β-arrestin1 finger loop may be inserted into the bundle intracellularly close to the TMH7-H8 elbow area [51,52,55,59]. Therefore, the sequence for the CB1 peptide encompasses the TMH7-H8 region, located at the intracellular side of the CB1 receptor. As in the case of β-arr1^63-76^, the specific peptide sequence (CB1^391-409^; Figure 1) was selected as the shortest peptide with higher α-helical propensity and solubility upon analysis using the AGADIR and protparam webservers [57,58] (Supplementary Table S2). 

To avoid effects of the ionisable amino and carboxylate groups, the N- and C-termini of the two peptides were acetylated and amidated, respectively. 

### 2.2. Structural Behavior of the Free CB1 and β-Arrestin1 Peptides

The conformation of the TMH7-H8 CB1 and β-arrestin1 peptides independently was examined in aqueous solution, in the presence of 30% of trifluoroethanol (TFE), a secondary structure enhancer [60], and using zwitterionic detergent micelles (dodecylphosphocholine, DPC) as membrane mimics.

**Figure 2:**
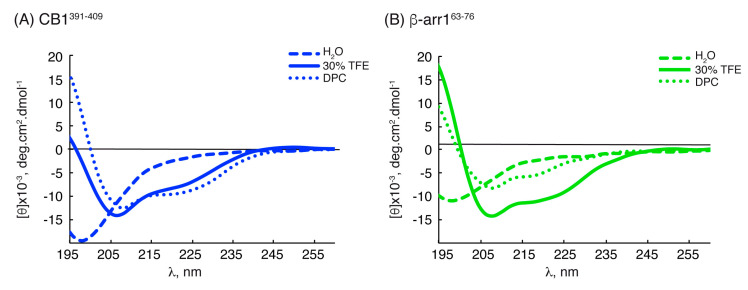
CD spectra of CB1^391-409^ (**A**) and β-arr1^63-76^ (**B**) in H_2_O (dashed line), TFE (continuous line) and DPC (dotted line) (30 mM). Data were collected at 5 °C, pH 5.5 and 50 µM peptide concentration.

We firstly characterized the structural behavior of the two peptides using circular dichroism (CD). As depicted in Figure 2, the CD spectra of the two peptides in water solution showed a minimum at about 197 nm, which indicated that they were mainly random coils, whereas they tended to form helical conformations in the presence of TFE or DPC micelles, as shown by the observed maximum below 195 nm and the minima at 208 nm and 222 nm. The helix percentages estimated from the ellipticity at 222 nm (**[θ]^222nm^**) are given in Table 1.

To gain further structural information, the peptides were characterized using NMR. The NMR spectra of the two peptides were fully assigned in the three experimental conditions, i.e., aqueous solution, in the presence of TFE and in DPC micelles (chemical shifts are reported in the supplementary material: Tables S3–S8). 

**Figure 3:**
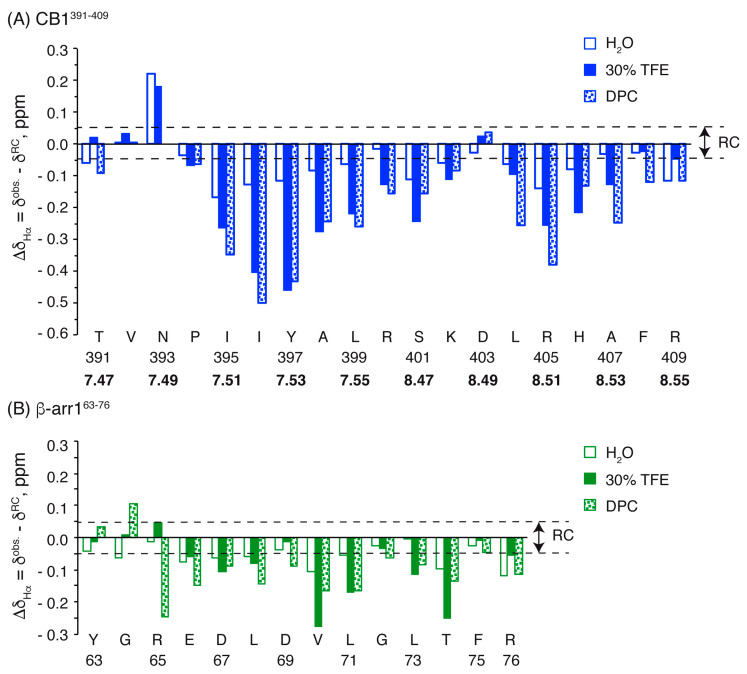
Δδ_Hα_ values plotted as a function of residue number for CB1^391-409^ (**A**) and β-arr1^63-76^ (**B**) in H_2_O (open bars), TFE (filled bars) and DPC (dotted bars) (30 mM). In all cases pH 5.5 and 25 °C. Dashed lines indicate the random coil (RC) range (|Δδ_Hα_| ≤ 0.05 ppm). In the case of CB1^391-409^, the Ballesteros and Weinstein numbering is shown in bold.

Most residues of the two peptides show negative Δδ_Hα_ and positive Δδ_Cα_ values (Figure 3 and Figure S3), which are large in magnitude in TFE and DPC micelles, and small in aqueous solution. In agreement with the CD data, this indicates that the peptides form helical structures in TFE and DPC, and have only a low helical tendency in aqueous solution. A detailed examination of the profiles showed that CB1^391-409^ presents two helical regions (P394-K402 and L404-F408), separated by the residue D403, which showed positive Δδ_Hα_ values in TFE and DPC (Figure 3), and negative Δδ_Cα_ values in aqueous solution and in DPC (Figure S3). The helical region in the β-arr1^63-76^ peptide extends from E66 to T74 in aqueous solution and in TFE, and from R65 to T74 in DPC. The percentages of helical populations estimated from Δδ_Hα_ and Δδ_Cα_ are given in Table 1.

Further evidence about the helix formation in the two peptides came from the sets of NOEs present in TFE and DPC, which included those characteristic of helical structures, i.e., sequential NN(i,i+1), and the nonsequential αN(i,i+3), and αβ(i,i+3). Examples of these NOEs are shown in the Supplementary Figures S1 and S2.

**Figure 4:**
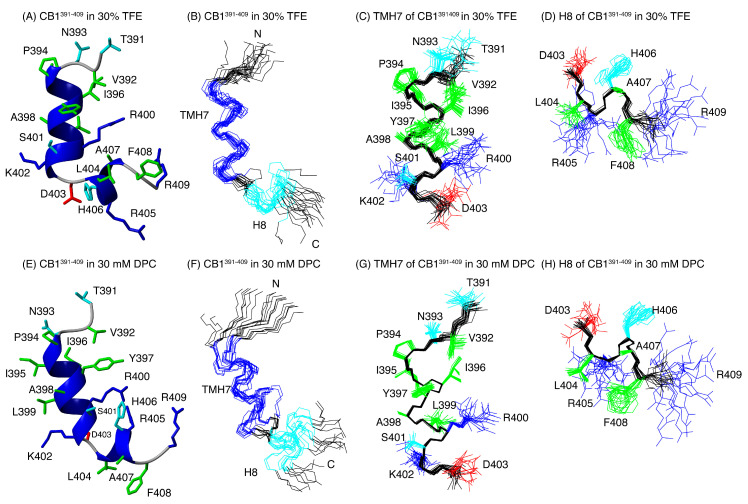
CB1^391-409^ structure in 30% TFE (**A**–**D**) and in DPC micelles (**E**–**H**). Ribbon representation of the lowest target conformer of the structural ensemble (**A**,**E**). Overlay of the backbone atoms of the 20 lowest target function conformers (**B**,**F**). Overlay of residues 394-402 (helix TMH7; panels (**C**,**G**)) Overlay of residues 403-407 (helix H8; panels (**D**,**H**)). In panels (**A**–**E**), the backbone atoms of helices TMH7 and H8 are coloured in blue. The side-chains are shown in panels (**F**–**H**). The aliphatic and aromatic side chains are displayed in green, the Glu and Asp side chains in red, the Arg and Lys side chains in blue, and the Asn, His, Thr, and Ser side chains in cyan.

**Figure 5:**
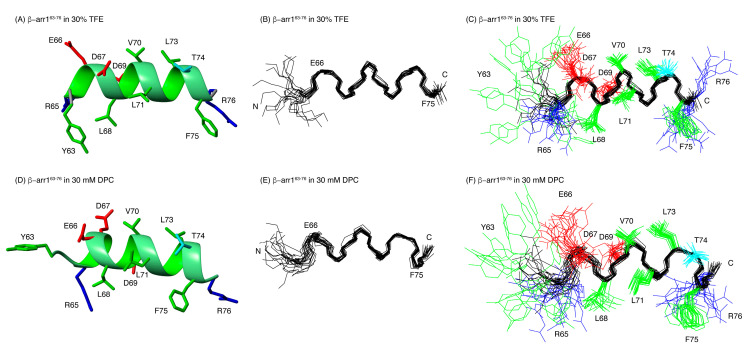
NMR structure of β-arr1^63-76^ in 30 % TFE (**A**–**C**) and in DPC micelles (**D**–**F**). Ribbon representation of the lowest target conformer of the structural ensemble (**A**,**D**). Overlay of the backbone atoms of the 20 lowest target function conformers (**B**,**E**). The side-chains are shown in panels (**A**,**C**–**E**). The aliphatic and aromatic side chains are displayed in green, the Glu and Asp side chains in red, the Arg and Lys side chains in blue, and the Thr side chains in cyan.

The preferred structures of the two peptides were calculated on the basis of distance and angle restraints derived, respectively, from the NOEs and the chemical shifts measured in TFE and in DPC and using the program CYANA (see Materials and Methods). The quality of the resulting structures is good (see Ramachandran plots at Supplementary Figure S4) and they are well defined (see RMSD values in Table S12). Figure 4 and Figure 5 illustrate overlays of the 20 lowest target function conformers for CB1^391-409^ and β-arr1^63-76^ peptides, as well as a representative conformer of the ensemble. In agreement with the qualitative analysis of Δδ_Hα_ and Δδ_Cα_ profiles, CB1^391-409^ in both TFE and DPC exhibits two helical regions, i.e., a long helix extending residues P394 to K402 and a short one spanning residues L404 to F408 (Figure 3A and Figure 4). The angle between the two helical regions shows certain variability among the conformers within the structural ensembles, but they are approximately perpendicular each other (94° ± 15° in TFE; 75° ± 30° in DPC; Figure 4) as in the crystalline structure of free CB1 (97° in PDB ID: 5XRA). TMH7, which ends at residue L399 in crystalline full-length CB1 receptor, extends up to residue K402 in the CB1^391-409^ peptide both in TFE and in DPC. This result is in agreement with the previously reported structure for another CB1-derived peptide containing the same region [61]. Tyukhtenko and coworkers studied the structure of the TMH7-H8 span (CB1^377-416^) obtaining a lengthy hydrophobic α-helical segment and a short amphipathic α-helix (H8) orthogonally oriented to TMH7. 

Our structural studies demonstrated that the β-arr1^63-76^ peptide also formed helical conformations in DPC and TFE (Figure 5). In agreement with our observations, various studies have indicated that in its activated state, the β-arrestin finger loop adopts helical conformations [55,56,62]. However, it is important to note that conformational plasticity of the finger loop was observed in previously reported GPCR/arrestin complexes [51,52,53,54,55]. While in the rhodopsin/arrestin complexes the finger loop forms a helical domain [54,55], in the recently solved muscarinic 2 receptor/arrestin complex [53], the finger loop adopts an extended loop configuration. This suggests that it can be ordered in different conformations or adopt diverse relative orientations in order to enable the recognition of a wide variety of GPCRs.

### 2.3. Characterization of the CB1 and β-Arrestin1 Interface

**Figure 6:**
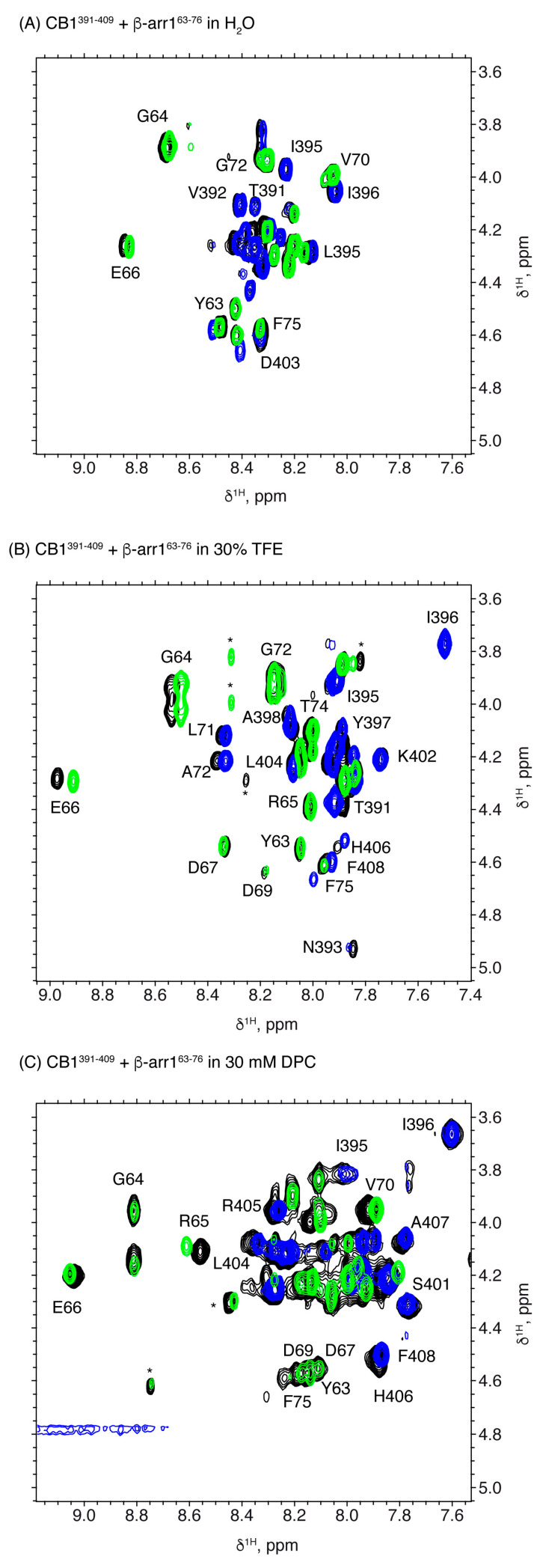
Overlay of selected regions of 2D ^1^H,^1^H TOCSY spectra for CB1^391-409^+ β-arr1^63-76^ (black contours), CB1^391-409^ (blue contours) and β-arr1^63-76^ (green contours) in water (**A**), in 30 % TFE (**B**) and in DPC micelles (**C**). In all cases pH 5.5 and 25 °C.

In order to elucidate whether CB1^391-409^ and β-arr1^63-76^ peptides are prone to interact, we acquired NMR spectra of the peptide mixture in the same conditions as for the isolated peptides. All the residues in the mixtures were unequivocally assigned (Supporting Information Tables S9–S11). As seen in the spectral regions shown in Figure 6 (see also Figures S5–S7), some cross-peaks are shifted in the spectra of the peptide mixture relative to the isolated peptides in the three examined experimental conditions. This result provides evidence that these two short peptides by themselves are able to interact each other.

**Figure 7:**
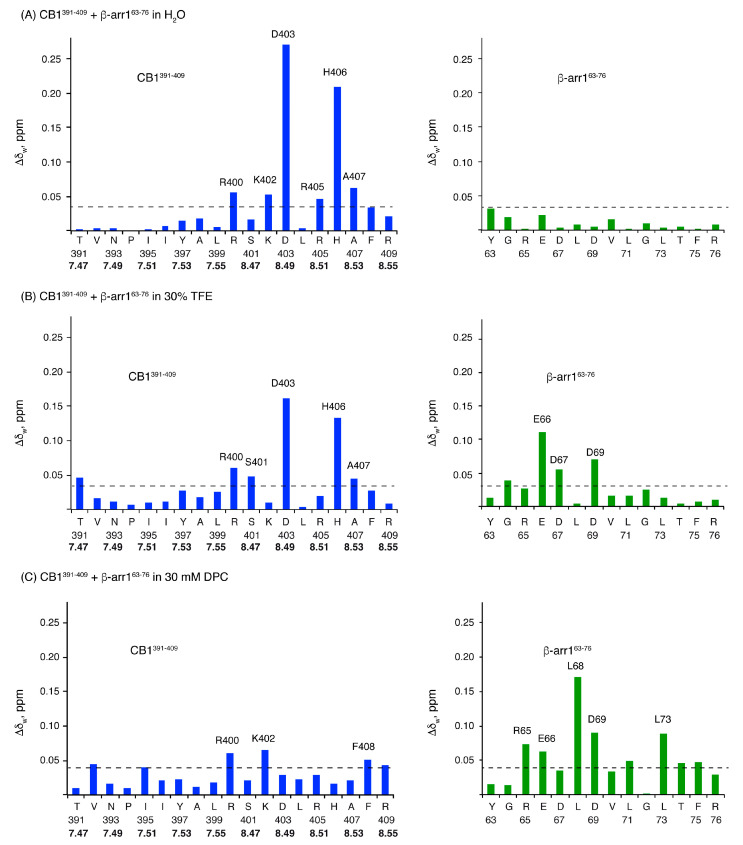
Weighted chemical shift differences (Δδ_w_, ppm) plotted as a function of peptide sequence for CB1^391-409^ (blue bars) and β-arr1^63-76^ (green bars). Δδ_w_ values are calculated as Δδ_w_=[(Δδ_Hαinteraction_)^2^+(Δδ_NHαinteraction_)^2^+((Δδ_Cαinteraction_)^2^/4)]^1/2^, where Δδ_Hαinteraction_, Δδ_NHαinteraction_ and Δδ_Cαinteraction_ are the chemical shift differences of the corresponding nuclei for the free peptides and in the mixture in H_2_O (**A**), 30% TFE (**B**) or 30 mM DPC (**C**) at pH 5.5 and 25 °C. In the case of CB1^391-409^, the Ballesteros and Weinstein numbering is shown in bold.

In aqueous solution, some cross-peaks belonging to CB1^391-409^ showed significant differences in the mixture relative to the isolated peptide (the most affected residues are D403 and H406; Figure 7A), whereas those of β-arr1^63-76^ were hardly affected (Figure 7A). This suggests that the interaction of these two peptides in aqueous solution requires some structural rearrangement in CB1^391-409^, but not in β-arr1^63-76^, whose conformational equilibrium remains mainly unaffected.

However, in TFE and DPC, significant weighted NMR chemical shift differences were observed in both β-arr1^63-76^ and CB1^391-409^ moieties when comparing the independent peptides with the mixture (Figure 7B,C). These changes are remarkable in residues D403 and H406 for CB1^391-409^ (which are also affected in aqueous solution; Figure 7A) and E66, D67 and D69 for β-arr1^63-76^ in TFE. DPC mixtures showed weighted NMR chemical shift differences in residues R400 and K402 of CB1^391-409^ and R65, E66, L68, D69 and L73 of β-arr1^63-76^. Table S13 summarizes the residues whose chemical shifts are affected upon interaction in each experimental condition. 

These results show that short model peptides encompassing residues belonging to the putative contact region in the model of the CB1/β-arrestin1 complex (Figure 1) are able to interact. Thus, these short sequences seem to contain enough information to recognize each other. However, how they interact seems to depend on the environment. The conformational rearrangement in CB1 is likely similar in water and in TFE, since the affected residues are essentially the same. But, upon CB1^391-409^ interaction, β-arr1^63-76^ suffers some reorganization in the presence of TFE, but hardly change in water. 

In the presence of DPC micelles, the two peptides might experience some conformational rearrangements, albeit somehow differently from those in water and TFE. These conformational changes might play a role in the CB1 β-arrestin 1 activation. 

As previously mentioned, structural rearrangements of the arrestin finger loop have already been observed depending on the environment, providing evidence for its necessary plasticity to couple to diverse GPCRs [51,53,55]. Table S14 displays the sequence diversity at the interface region of the GPCRs elucidated in complex with arrestins compared to CB1. This demonstrates the ability of the finger loop domain to conformationally adapt according to the interacting partner.

**Figure 8:**
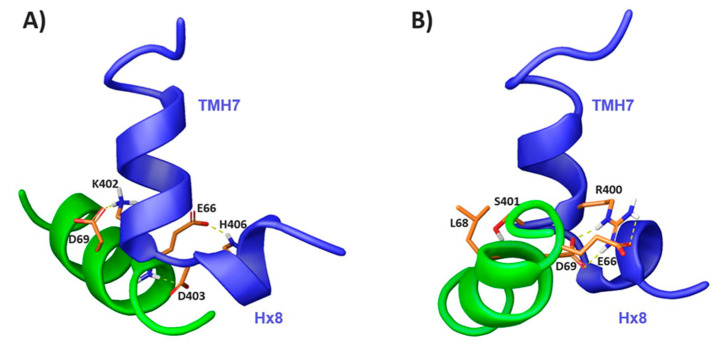
CB1^391-409^/β-arr1^63-76^ complex models in TFE (**A**) and DPC (**B**). Main interacting residues are displayed in orange and yellow dashed lines represent hydrogen bonds.

To visualize how the peptides contact each other and if they are reproducing the way of interaction of the full-length proteins, we proceeded to model the complexes. For that purpose, we used the Haddock-webserver introducing the structures calculated for the isolated peptides in TFE and in DPC as input. This program requires the definition of interacting residues defined as active in the docking interface. These are the amino acids whose resonances show changes in the peptide mixture for each condition (Figure 7). Figure 8 depicts a representative model of the CB1^391-409^/β-arr1^63-76^ complex in each condition selected from the cluster with the best Haddock docking score. These models exhibit the different rearrangement of the peptides, depending on the environment. While in DPC, β-arr1^63-76^ is almost parallel to the H8 portion of CB1^391-409^, in TFE, β-arr1^63-76^ sits perpendicularly to both CB1^391-409^ helical domains. Main interactions involved in the interface of the TFE complex model include hydrogen bonds between K402 and D69, H406 and E66, and the interaction formed by E66 backbone with D403 side chain (Figure 8A). The DPC complex model is mainly stabilized by hydrogen bond interactions of R400 with E66 and D69, and L68 backbone with S401 side chain (Figure 8B). In both conditions, there is also a reduction of solvent accessible surface area (ASA) upon complex formation in β-arr1 residues E66 and D69. These divergences in the peptide rearrangement, depending on the environment, could be due to the conformational plasticity of the studied region. This is in agreement with the structural diversity observed in the GPCR/arrestin finger loop interface of the reported complexes [51,52,53,54,55].

It is worth noting that in the few GPCR-arrestin complexes reported thus far (none of them with CB1 receptors), residues in analogous positions of the GPCR and arrestin play a key role in their interface. For instance, residue D69 in activated β-arrestin1 was shown to directly engage with the elbow region of the β1-adrenergic receptor in a recently elucidated complex [51].

## 3. Materials and Methods 

### 3.1. Chemicals and Peptides

The deuterated compounds and solvents [D_38_]-dodecylphosphocholine (DPC) (98%), [D_3_]-2,2,2-trifluoroethanol (TFE) (99%) and D_2_O (99.9%) were purchased from Cambridge Isotope Laboratories (USA). Deuteration percentages are given in parentheses. 

Designed peptides, with acetylated amino termini and amidated carboxylate ends, were synthesized on demand by CASLO ApS (Denmark). Solid-phase synthetic procedures along with reverse-phase HPLC purification yielded the desired peptides with the indicated purities:

- *CB_1_ peptide* (CB1^391-409^; Ac-TVNPIIYALRSKDLRHAFR-NH_2_): HPLC: t_R_ = 17.2 min; 95.3% (gradient: 18-36% B in 23 min; buffer A: 0.05% TFA + 2% CH_3_CN; buffer B: 0.05% TFA + 90% CH_3_CN). MALDI-TOF: Theoretical MW = 2311.74; Found [M+H]^+^ = 2312.17.
- *β-arrestin1 peptide* (β-arr1^63-76^; Ac-YGREDLDVLGLTFR-NH_2_): HPLC: t_R_ = 7.9 min; 95.0% (gradient: 30-44% B in 14 min; buffer A: 0.05% TFA + 2% CH_3_CN; buffer B: 0.05% TFA + 90% CH_3_CN). MALDI-TOF: Theoretical MW = 1694.93; Found [M+H]^+^ = 1696.22.

### 3.2. Peptide Numbering

The absolute sequence number of peptide residues was used throughout the article. The Ballesteros−Weinstein numbering system for GPCR amino acid residues is provided in Figure 1 to facilitate the identification of key GPCR positions [63]. 

### 3.3. CD Spectroscopy

CD spectra of the peptides were recorded using a J-815 spectropolarimeter (JASCO, Groß-Umstadt, Germany). Stock solutions of each peptide were prepared at a nominal concentration of 1mg mL^−1^ in milliQ-water. Samples in DPC micelles were prepared by dilution of a 30 mM DPC stock solution in milliQ-water. In both conditions, peptide final concentrations were 50 μM. Measurements were recorded at 5 °C in a quartz glass cells (Suprasil, Hellma, Müllheim, Germany) of 1 mm path length, between 260 and 190 nm at 0.1 nm intervals. 

Isothermal spectra for these samples were acquired at a scan speed of 50 nm min^−1^ with a response time of 4 s and 1 nm bandwidth. Over four scans were averaged for each sample and for the baseline of the corresponding peptide-free sample. Upon baseline correction, CD data were processed with the adaptive smoothing method integrated in the Jasco Spectra Analysis software. CD data are given in molar ellipticity units ([θ], deg cm^2^ dmol^−1^) for the isolated peptides and ellipticity units (θ, mdeg) for mixtures. 

Estimations of the helix percentages for the free peptides were obtained from the experimental [θ] value at 222 nm ([θ]^222nm^, deg.cm^2^.dmol^−1^) by applying Equation (1):

(1) $$\%\;helix=\frac{-{\left[\theta \right]}^{222nm}+3000}{39000}$$

### 3.4. NMR Studies

#### 3.4.1. Sample Preparation

Lyophilized peptides were dissolved in a final volume of 0.5 mL of solvent; that is, H_2_O/D_2_O (9:1 ratio by volume), pure D_2_O, 30%[D_3_]-TFE/70% H_2_O/D_2_O (9:1), and 30 mM [D_38_]-DPC in H_2_O/D_2_O (9:1, v/v) or in pure D_2_O. Final peptide concentrations were of 1.0 mM in all the NMR samples. The pH was measured using a glass micro-electrode and adjusted to 5.5 by addition of NaOD or DCl. Samples were placed in 5 mm NMR tubes and 2 μL of sodium 2,2-dimethyl-2-silapentane-5-sulfonate (DSS) were added as internal reference for ^1^H chemical shifts.

#### 3.4.2. Spectra Acquisition

A Bruker Avance-600 spectrometer (600 MHz) was used to record NMR spectra. Standard techniques were used to acquire 2D spectra: COSY (phase sensitive correlated spectroscopy), TOCSY (total correlated spectroscopy), and NOESY (nuclear Overhauser enhancement spectroscopy). Water signal suppression was achieved by presaturation or Watergate [64]. Mixing times of 60 ms were used to record the TOCSY spectra while 150 ms were used for the NOESY. ^1^H-^13^C HSQC (heteronuclear single quantum coherence spectroscopy) were acquired at ^13^C natural abundance. The IUPAC-IUB recommended ^1^H/^13^C chemical shift ratio was employed to indirectly referenced the ^13^C chemical shifts [65]. Depending on the experimental conditions, peptide samples were tested at 5 and/or 25 °C. Data processing was accomplished using the TOPSPIN software (Bruker Biospin, Karlsruhe, Germany).

#### 3.4.3. Spectra Assignment

The well-established sequential methodology based on homonuclear spectra [66] was used to assign the NMR spectra of each sample. This was done using the tools provided by the NMR assignment program SPARKY (NMRFAM-Sparky version 1.4) [67]. ^13^C resonances were assigned based on the cross-peaks observed in the ^1^H-^13^C-HSQC spectra. ^1^H and ^13^C chemical shifts are listed in the Supporting Tables S3–S11 and been deposited at the BioMagResBank (http://www.bmrb.wisc.edu) with accession codes BMRB ID: 50372-50377 and 50382-50384.

#### 3.4.4. Estimation of Helix Populations

Helix populations were obtained from the H_α_ and ^13^C_α_ chemical shifts as previously described [68]. The errors in the populations estimated from the H_α_ and ^13^C_α_ chemical shifts are approx. 3 and 7%, respectively, assuming experimental errors of 0.01 and 0.1 ppm in the measurement of ^1^H and ^13^C chemical shifts.

### 3.5. Structure Calculation

Structure calculations of the studied peptides were performed using the iterative procedure for automatic NOE assignment integrated in the CYANA 3.97 program [69]. The CYANA algorithm uses an iterative process having seven cycles, in which NOEs are automatically assigned by a probabilistic treatment, and structures are calculated from them. The program computes 100 conformers per cycle, minimizing the 20 structures with the lowest target functions. 

The assigned chemical shifts, the NOE integrated cross-peaks (as observed in the NOESY spectra) and the φ and ψ dihedral angle restraints (obtained using TALOSn webserver [70]) were used as experimental input data for structure calculation (Table S12).

The Maestro software, integrated in the Schrödinger 2018 package (Schrödinger Inc., Portland, OR), and the MOLMOL program [68] were used to visualize and examine the final ensembles of the 20 lowest target function conformers. The protein preparation wizard implemented in Maestro was used to assess their quality and ensure structural correctness.

### 3.6. NMR-Driven Docking

A model of the CB1/β-arrestin1 interaction complex was built using the Haddock-webserver (http://milou.science.uu.nl/services/HADDOCK2.2/) [71,72]. The PDB coordinates determined herein for the solution structure of each peptide were used as input. The active residues in the docking interface were those whose NMR signals in the free peptides and in the mixture showed significant differences. These active residues guide the search for the best interacting way of the two input molecules. Haddock follows a rigid body energy minimization to cluster the complex models. In this way, the 200 complex models with lowest energy values were clustered and then refined using semiflexible docking and explicit water solvation. Representative complexes were those showing the best Haddock docking scores.

## 4. Conclusions

In the search of improved therapeutics targeting CB1 receptors, biased ligands are currently a major hope and challenge for avoiding undesired effects while optimizing the beneficial outcome. The design of these compounds clearly depends on an in-depth structural understanding of the GPCR-effector mechanism.

Since the G-protein interaction to CB1 has already been extensively explored [49,50], in this work, we aim to provide insights into the CB1/β-arrestin1 interface. This arrestin isoform was chosen due to the fact that it can provoke G protein-independent activation of the ERK signaling pathway [27]. For this purpose, based on reported complexes of β-arrestin with other GPCRs, we identified a putative binding region of the β-arrestin1 finger loop in CB1_._ We characterized the structure of the CB1 TMH7-H8 elbow region and the β-arrestin1 finger loop, as well as their interaction using model peptides. The structural data obtained using CD and NMR studies indicated that both peptides had a slight tendency to be helical in aqueous solution, with the helical conformations being greatly stabilized in the presence of TFE and DPC micelles. It should be noted that TFE is a secondary structure enhancer, which has been shown to stabilize both helices and β-sheets [60,73] and that amphipathic structures, helical or not, seem to be favored in DPC micelles [74]. NMR characterization of CB1^391-409^ confirmed the formation of two distinct helical motifs orthogonally oriented mimicking their corresponding region at TMH7 and H8. Therefore, this short peptide is able to maintain, at least partially, the structure of the full-length protein. Concerning β-arr1^63-76^ finger loop model peptide, it tended to adopt helical conformations, which is in agreement with some of the reported activated β-arrestins [54,55,56,62], but not with others in which the finger loop is not helical [51,53]. The fact that the helix stability of the β-arr1^63-76^ finger loop is low might be related to the plasticity of this region to adopt diverse structures in order to adapt to its partner. So, this short peptide would be reproducing the structural behavior of the full-length protein.

More interestingly, as observed in the peptides mixture spectra, residues at the TMH7-H8 elbow can interact with the domain of the β-arrestin1 finger loop. This structural information is in agreement with the few previously reported structures of β-arrestins in complex with other class A GPCRs such as the rhodopsin or the β1-adrenergic receptors [51,52,53,55]. Structural changes at this intracellular receptor region may suggest that the extracellular domain of the TMH1-2-7 region is involved in ligand binding of CB1 β-arrestin1 biased ligands. Therefore, this information may provide further insights into the design of novel CB1 molecules with optimized therapeutic outcomes. 

In the context of the intricate GPCR signaling, β-arrestin pathway inhibition can help in elucidating specific pharmacological outcomes through other effector proteins. So far, β-arrestin blockage has been mainly accomplished through the use of siRNA-mediated knockdown or CRISPR/Cas9 methods, due to the lack of other analytical tools [75]. Our studied β-arrestin1 finger loop peptide β-arr1^63-76^ demonstrated the ability to interact with the CB1 TMH7-H8 elbow region. Therefore, this peptide could represent a useful pharmacological tool to block β-arrestin1 binding to this cannabinoid receptor under particular conditions, facilitating the study of its intriguing signaling. 

In summary, our results show that short peptides encompassing the sequences of the TMH7-H8 intracellular domain and the β-arrestin1 finger loop tend to adopt the structural features of the full-length proteins, and are able to interact each other in a way that parallels the putative CB1/β-arrestin1 interface, as deduced from other GPCR/arrestin complexes. Apart from providing structural insights into the CB1/β-arrestin1 recognition, our findings might open a way towards the selective blocking of the β-arrestin1 pathway. Further studies using CB1^391-409^ and β-arr1^63-76^ mutants and considering TMH6 and intracellular loops will be developed in order to fully unravel the key molecular features involved in CB_1_ recognition of the finger loop domain of β-arrestin1, which would evidently also be understood if the structure of the whole CB1/β-arrestin1 complex is determined in the future.

## Figures and Tables

**Table 1:** Percentages of helical populations for CB1^391-409^ and β-arr1^63-76^ peptides from CD and NMR data (see Materials & Methods) in H_2_O, TFE and DPC at 25 °C.

Peptide	Conditions	[θ]^222nm^, deg.cm^2^.dmol^−1^	% Helix ^[a]^ from [θ]^222nm^	Helix Length	Av. Δδ_H__α_, ppm	% α-Helix from Δδ_Hα_	Av. Δδ_C__α_, ppm	% α-Helix from Δδ_Cα_	Av. % Helix^[c]^
CB1^391-409^	H_2_O	−2178.68	13	P394-K402	−0.09 ^[b]^	22 ^[b]^	+0.48 ^[b]^	16 ^[b]^	19 ± 3 ^[b]^
				L404-F408	−0.07 ^[b]^	18 ^[b]^	+0.33 ^[b]^	11 ^[b]^	14 ± 4 ^[b]^
	TFE	−7931.79	28	P394-K402	−0.24	62	+2.50	81	71 ± 9
				L404-F408	−0.14	37	+1.52	49	43 ± 6
	DPC	−9415.07	32	P394-K402	−0.25	64	+2.01	65	64 ± 1
				L404-F408	−0.23	58	+1.69	54	56 ± 2
β-arr1^63-76^	H_2_O	−1750.71	12	E66-T74	−0.06 ^[b]^	16 ^[b]^	+0.56 ^[b]^	18 ^[b]^	17 ± 2 ^[b]^
	TFE	−10229.3	34	E66-T74	−0.12	31	+1.79	58	45 ± 13
	DPC	−3626.36	17	R65-T74	−0.13	34	+1.22	39	37 ± 3

^[a]^ Notice that CD-estimated helix percentages are an average for all the peptide residues, whereas NMR-estimated helix percentages are for the residues within the helix. ^[b]^ Values measured at 5 °C. ^[c]^ Reported errors are standard deviations for the mean of the percentages obtained from the Δδ_Hα_ and Δδ_Cα_ values.

## Supplementary Materials

Supplementary materials can be found at https://www.mdpi.com/1422-0067/21/21/8111/s1. Figure S1. Selected regions of the 2D ^1^H, ^1^H NOESY of CB1^391-409^ in TFE and in DPC micelles. Figure S2. Selected regions of the 2D ^1^H, ^1^H NOESY of β-arr1^63-76^ in TFE and in DPCmicelles. Figure S3. ΔδCα conformational shifts as a function of peptide sequence for CB1^391-409^ and β- arr1^63-76^ peptides. Figure S4. Ramachandran plots for the NMR structures of CB1^391-409^ and β-arr1^63-76^ in 30 % TFE and in DPC micelles. Figure S5. Overlay of selected regions of 2D ^1^H, ^1^H TOCSY spectra for the mixture of CB1^391-409^ plus β-arr1^63-76^, and for the isolated CB1^391-409^ and β-arr1^63-76^ in aqueous solution at 5 oC. Figure S6. Overlay of selected regions of 2D ^1^H, ^1^H TOCSY spectra for the mixture of CB1^391-409^ plus β-arr1^63-76^, and for the isolated CB1^391-409^ and β-arr1^63-76^ in 30% TFE at 25 oC. Figure S7. Overlay of selected regions of 2D ^1^H, ^1^H TOCSY spectra for the mixture of CB1^391-409^ plus β-arr1^63-76^, and for the isolated CB1^391-409^ and β-arr1^63-76^ in DPC micelles at 25 oC. Table S1. β -arrestin1 finger loop peptide design. Table S2. CB1 TMH7-Hx8 peptide design Tables S3–S11. ^1^H, and 13C chemical shifts of CB1^391-409^ and β-arr1^63-76^ peptides under different experimental conditions. Table S12. Summary of structural statistics parameters for CB1^391-409^ and β-arr1^63-76^ peptides. Table S13. CB1^391-409^ and β-arr1^63-76^ residues whose chemical shifts are affected upon interaction. Table S14. Sequence alignment of GPCRs reported in complex with arrestins compared to CB1 at the studied TMH7-H8 region.

## Abbreviations

AC	Adenylyl cyclase
cAMP	Cyclic adenosine monophosphate
CB1	Cannabinoid receptor type 1
CD	Circular dichroism
COSY	Phase sensitive correlated spectroscopy
DPC	Dodecylphosphocholine
FL	Finger loop
pERK1/2	Extracellular signal-regulated kinase 1/2
GIRKs	G protein-coupled inwardly-rectifying potassium channels
GPCR	G protein-coupled receptor
GRKs	G protein-coupled receptor kinases
MAPK	Mitogen-activated protein kinases
NOESY	Nuclear Overhauser enhancement spectroscopy
PI3K	Phosphatidylinositide-3-kinase
TFE	Trifluoroethanol
TMH	Transmembrane helix
TOCSY	Total correlated spectroscopy
VGCC	Voltage-gated calcium channels
