# Supplementary material for: Structural Insights into β-arrestin/CB1 Receptor Interaction: NMR and CD Studies on Model Peptides
Source: Int J Mol Sci. 2020 Oct 30;21(21):8111. doi: 10.3390/ijms21218111 (PMC7662265; doi:10.3390/ijms21218111)
Supplement: Supplementary file 1 [file ijms-21-08111-s001.pdf]

Supporting Information for

Structural Insights into the β-arrestin/CB1 Receptor Interaction: NMR and CD Studies on Model Peptides

Paula Morales [1,2],* Marta Bruix [1] and M. Angeles Jiménez [1,]*

[1] Departamento de Química Física Biológica, Instituto de Química Física Rocasolano (IQFR-CSIC), Serrano 119, 28006 Madrid, Spain; marta.bruix.gmail.com (M. B.)

[2] Instituto de Química Médica (IQM-CSIC), Juan de la Cierva 3, 28006, Madrid, Spain;

* Correspondence: paula.morales@iqm.csic.es (P. M.)
majimenez@iqfr.csic.es (M.A.J.)

Summary of contents

List of figures:

Figure S1. Selected regions of the 2D ^{1}H,^{1}H NOESY of CB1$^{391\text{-}409}$ in TFE and in DPC micelles

Figure S2. Selected regions of the 2D ^{1}H,^{1}H NOESY of β-arr1$^{63\text{-}76}$ in TFE and in DPC micelles.

Figure S3. $\Delta\delta_{C\alpha}$ conformational shifts as a function of peptide sequence for CB1$^{391\text{-}409}$ and β-arr1$^{63\text{-}76}$ peptides.

Figure S4. Ramachandran plots for the NMR structures of CB1$^{391\text{-}409}$ and β-arr1$^{63\text{-}76}$ in 30 % TFE and in DPC micelles.

Figure S5. Overlay of selected regions of 2D ^{1}H,^{1}H TOCSY spectra for the mixture of CB1$^{391\text{-}409}$ plus β-arr1$^{63\text{-}76}$, and for the isolated CB1$^{391\text{-}409}$ and β-arr1$^{63\text{-}76}$ in aqueous solution at 5 ºC.

Figure S6. Overlay of selected regions of 2D ^{1}H,^{1}H TOCSY spectra for the mixture of CB1$^{391\text{-}409}$ plus β-arr1$^{63\text{-}76}$, and for the isolated CB1$^{391\text{-}409}$ and β-arr1$^{63\text{-}76}$ in 30% TFE at 25 ºC.

Figure S7. Overlay of selected regions of 2D ^{1}H,^{1}H TOCSY spectra for the mixture of CB1$^{391\text{-}409}$ plus β-arr1$^{63\text{-}76}$, and for the isolated CB1$^{391\text{-}409}$ and β-arr1$^{63\text{-}76}$ in DPC micelles at 25 ºC.

List of Tables:

Table S1. β–arrestin1 finger loop peptide design.

Table S2. CB1 TMH7-Hx8 peptide design

Tables S3-S11. ^{1}H, and ^{13}C chemical shifts of CB1$^{391\text{-}409}$ and β-arr1$^{63\text{-}76}$ peptides under different experimental conditions.

Table S12. Summary of structural statistics parameters for CB1$^{391\text{-}409}$ and β-arr1$^{63\text{-}76}$ peptides.

Table S13. CB1$^{391\text{-}409}$ and β-arr1$^{63\text{-}76}$ residues whose chemical shifts are affected upon interaction..

Table S14. Sequence alignment of GPCRs reported in complex with arrestins compared to CB1 at the studied TMH7-H8 region.

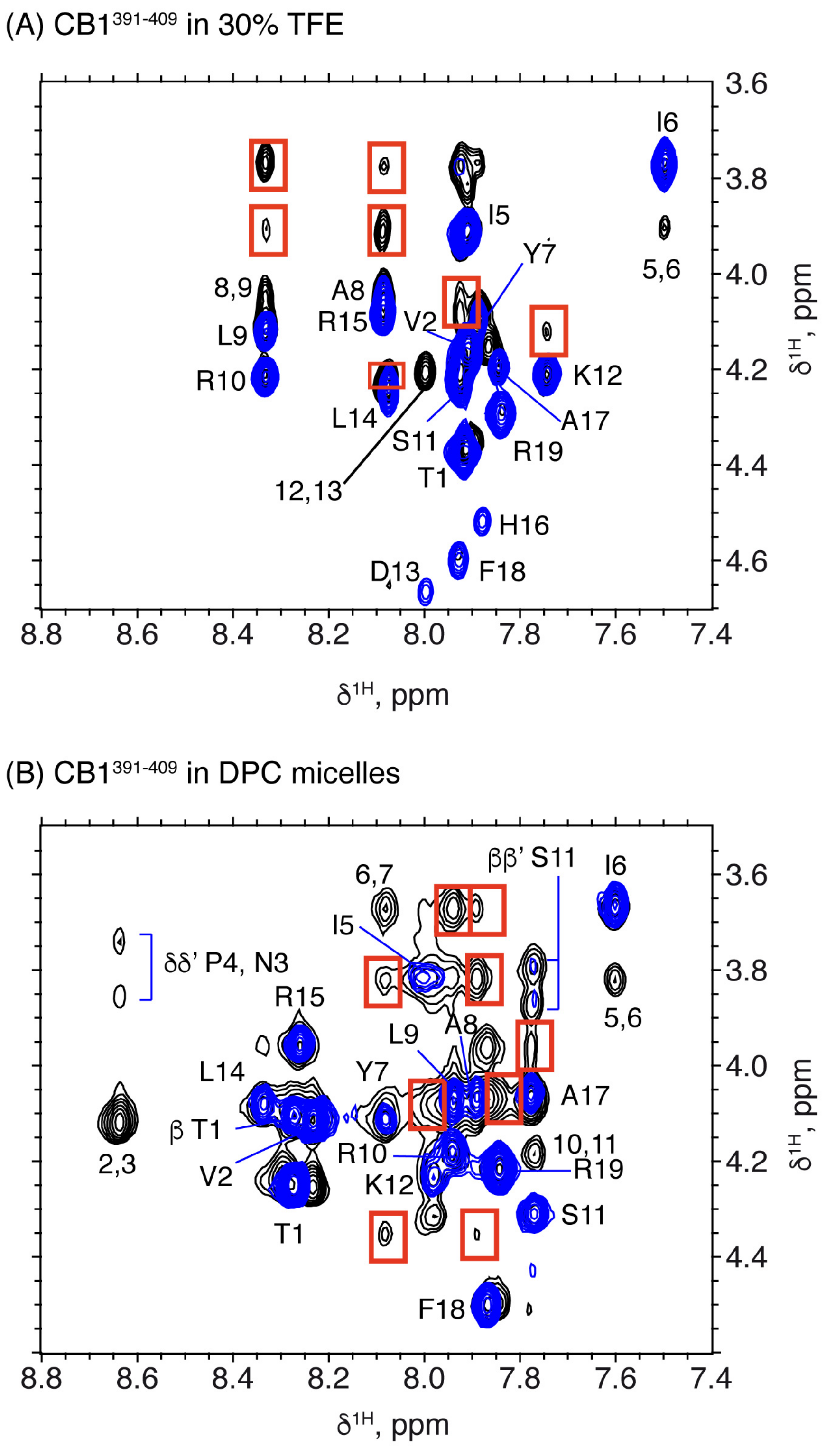

Figure S1. Overlay of the regions showing cross-peaks between HN amide and Hα protons for the 2D ^{1}H,^{1}H NOESY (black contours) and 2D ^{1}H,^{1}H TOCSY (blue contours) of CB1$^{391-409}$ in TFE (A) and in DPC micelles (B). TOCSY cross-peaks as well as many sequential NOE cross-peaks are labelled. To avoid label crowding relative residue numbers are used (see Table S2 for correspondence to absolute numbering). Non-sequential NOE cross-peaks are highlighted by red boxes. Sequential NOE cross-peaks are labelled indicating the numbers of the two residues separated by a comma.

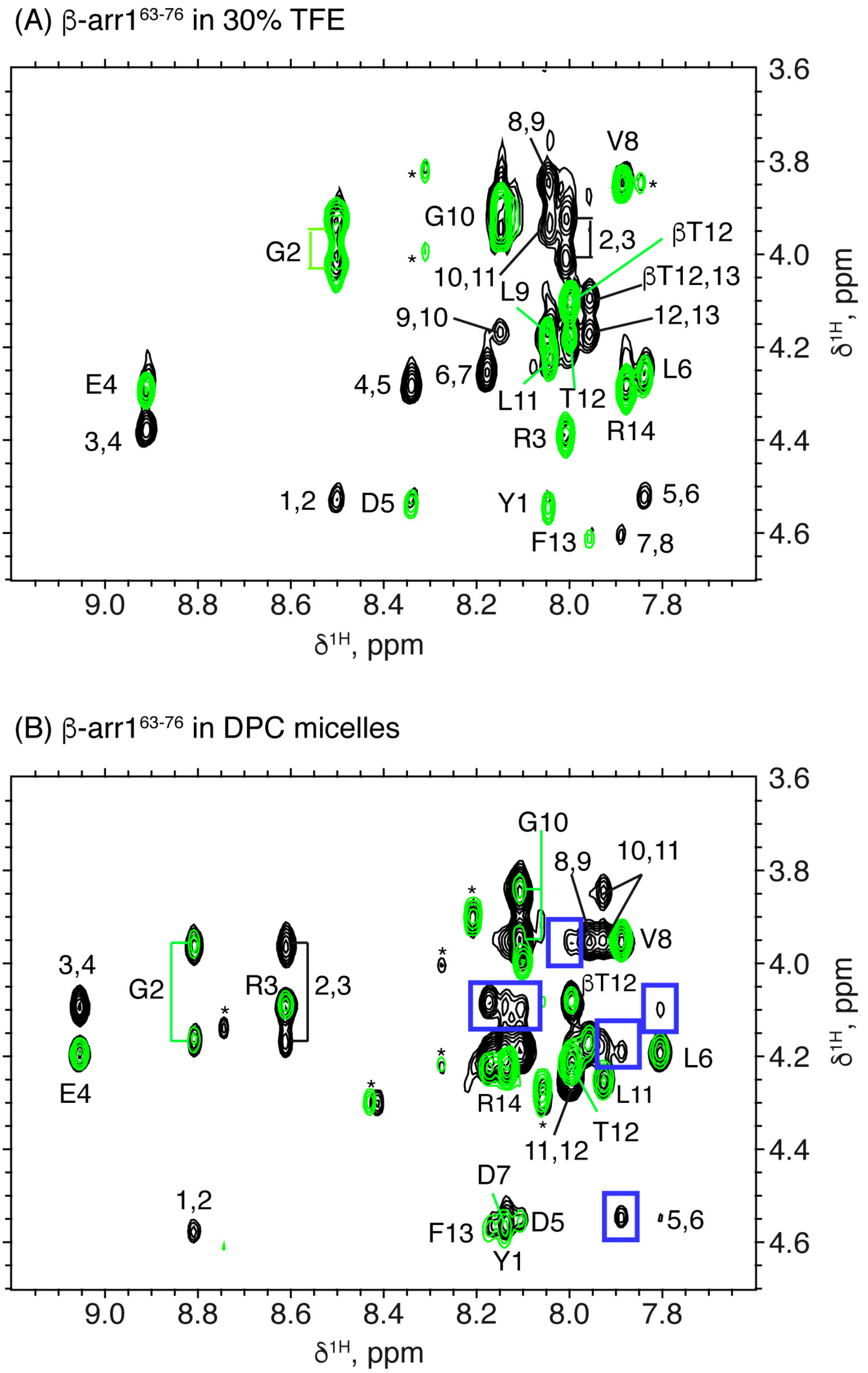

Figure S2. Overlay of the regions showing cross-peaks between HN amide and Hα protons for the 2D ^{1}H,^{1}H NOESY (black contours) and 2D ^{1}H,^{1}H TOCSY (green contours) of β-arr1$^{63\text{-}76}$ in in TFE (A) and in DPC micelles (B). TOCSY cross-peaks as well as many sequential NOE cross-peaks are labelled. Asterisks (*) indicate cross-peaks from impurities. To avoid label crowding relative residue numbers are used (see Table S1 for correspondence to absolute numbering). Non-sequential NOE cross-peaks are highlighted by blue boxes. Sequential NOE cross-peaks are labelled indicating the numbers of the two residues separated by a comma.

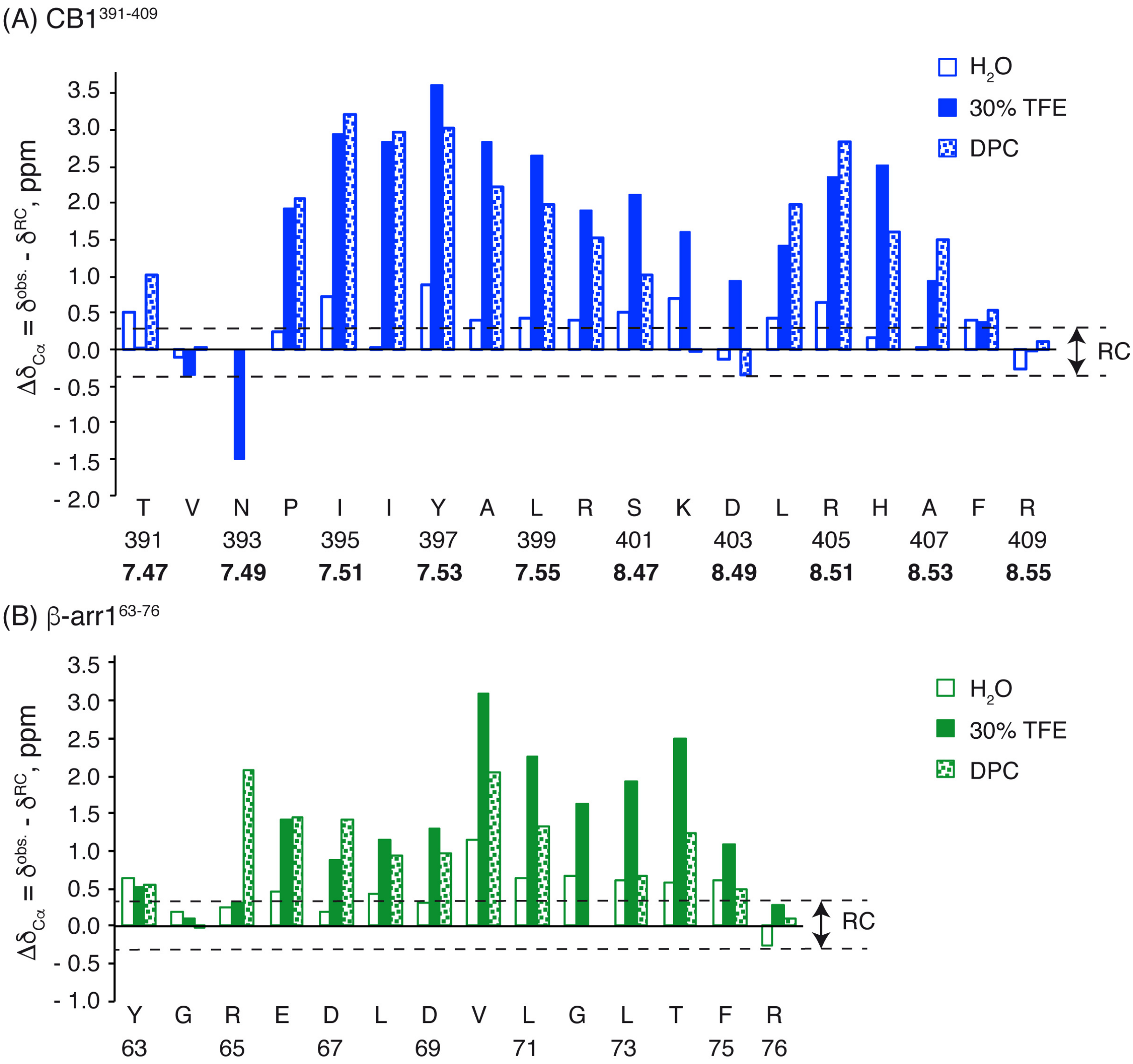

Figure S3. $\Delta\delta_{C\alpha}$ values plotted as a function of residue number for CB1$^{391\text{-}409}$ (A) and β-arr1$^{63\text{-}76}$ (B) in H_2O (open bars), 30% TFE (filled bars) and 30 mM DPC (dotted bars). In all cases pH 5.5 and 25°C. Dashed lines indicate the random coil (RC) range ($|\Delta\delta_{C\alpha}| \leq 0.4$ ppm). In panel A, the Ballesteros-Weinstein GPCR numbering is shown in bold.

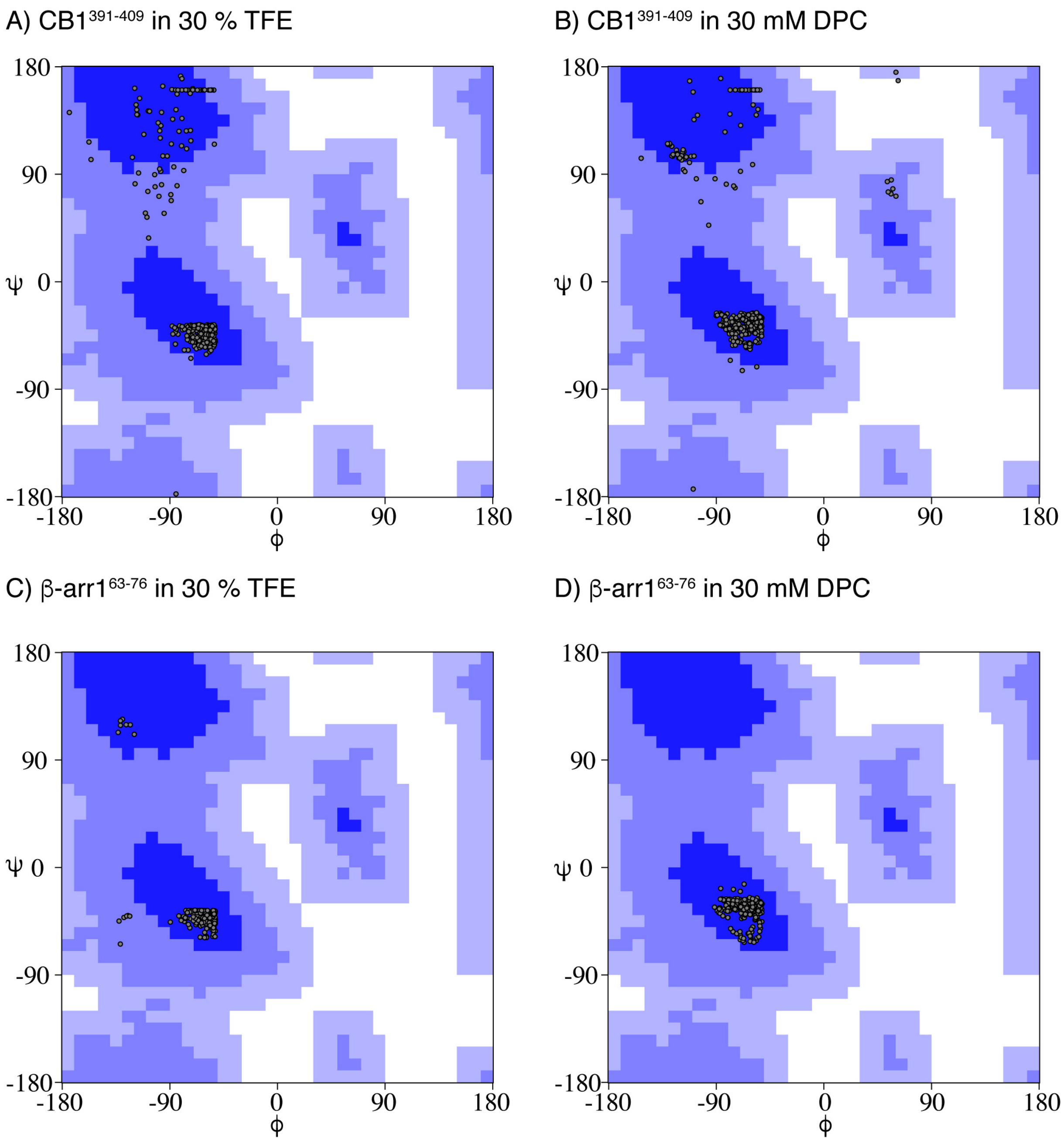

Figure S4. Ramachandran plots showing the ϕ, ψ angles (black dots) for NMR structural ensemble of the 20 lowest target function conformers of $CB1^{391\text{-}409}$ (A-B) and $\beta\text{-arr1}^{63\text{-}76}$ (C-D) in 30 % TFE and in DPC micelles. The percentages of residues in most favoured, additionally allowed, generously allowed and disallowed are listed in Table S12.

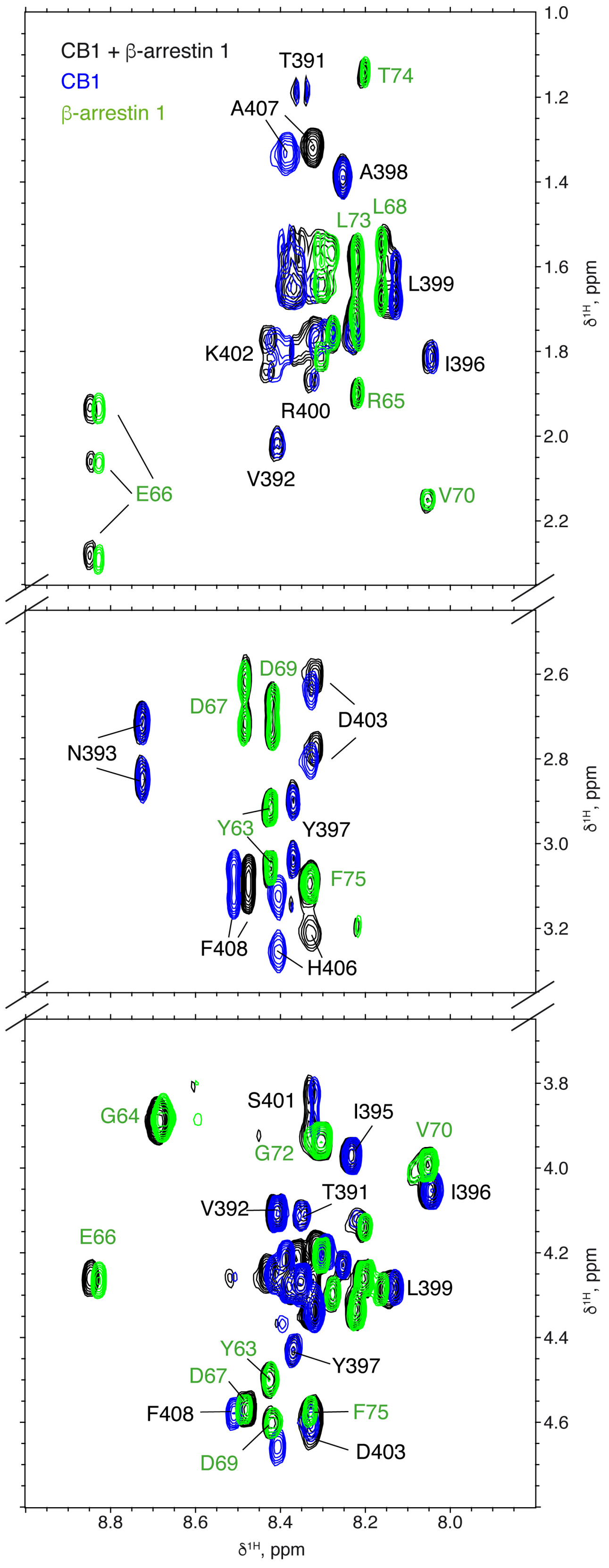

Figure S4. Overlay of selected regions of 2D ^{1}H,^{1}H TOCSY spectra for CB1$^{391\text{-}409}$ + β-arr1$^{63\text{-}76}$ (black contours), CB1$^{391\text{-}409}$ (blue contours) and β-arr1$^{63\text{-}76}$ (green contours) in aqueous solution at 5 ºC. Vertical is split to avoid showing regions without any cross-peak.

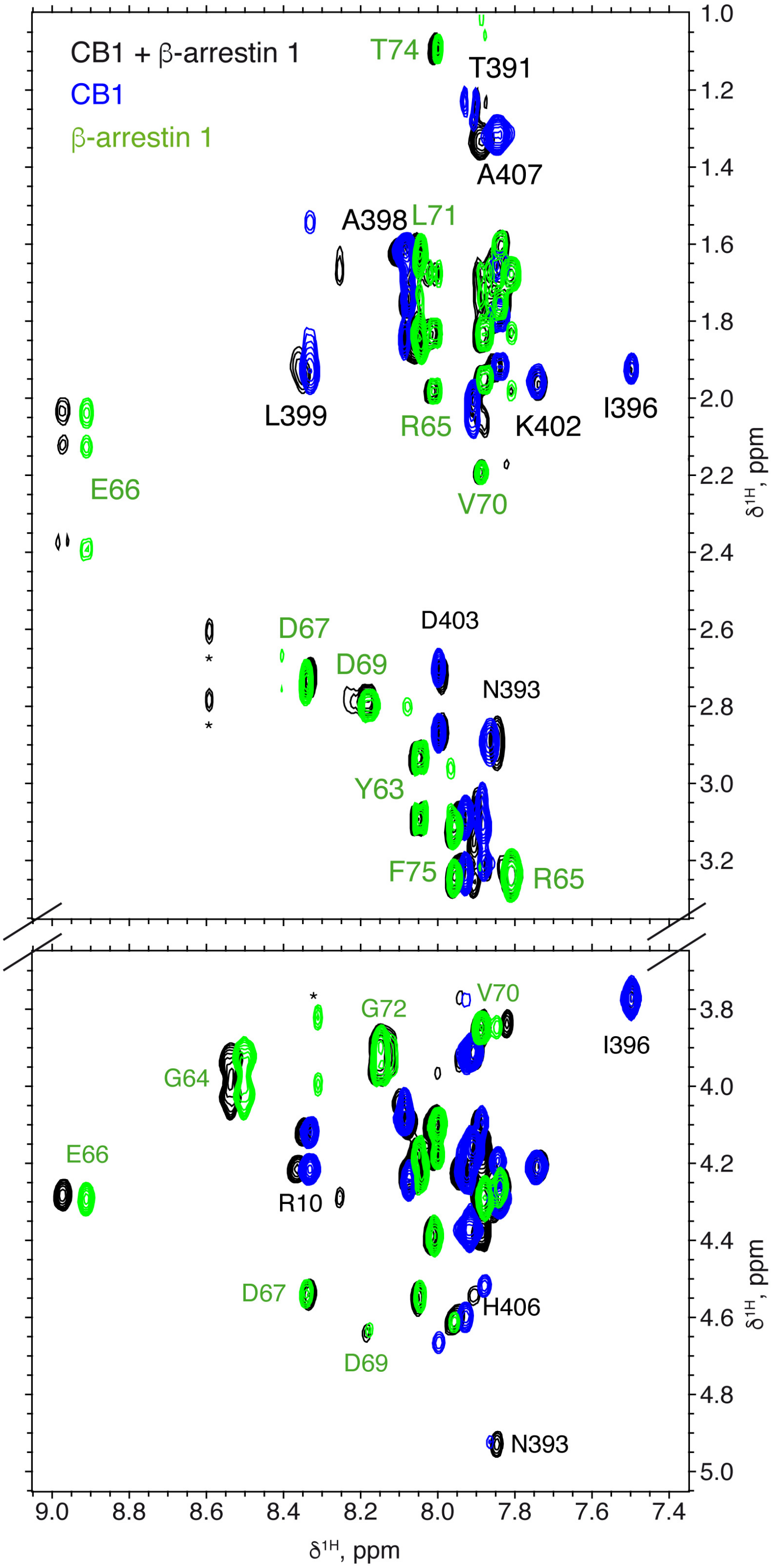

Figure S5. Overlay of selected regions of 2D ^{1}H,^{1}H TOCSY spectra for CB1$^{391-409}$ + β-arr1$^{63-76}$ (black contours), CB1$^{391-409}$ (blue contours) and β-arr1$^{63-76}$ (green contours) in 30% TFE at 25 ºC. Vertical is split to avoid showing regions without any cross-peak.

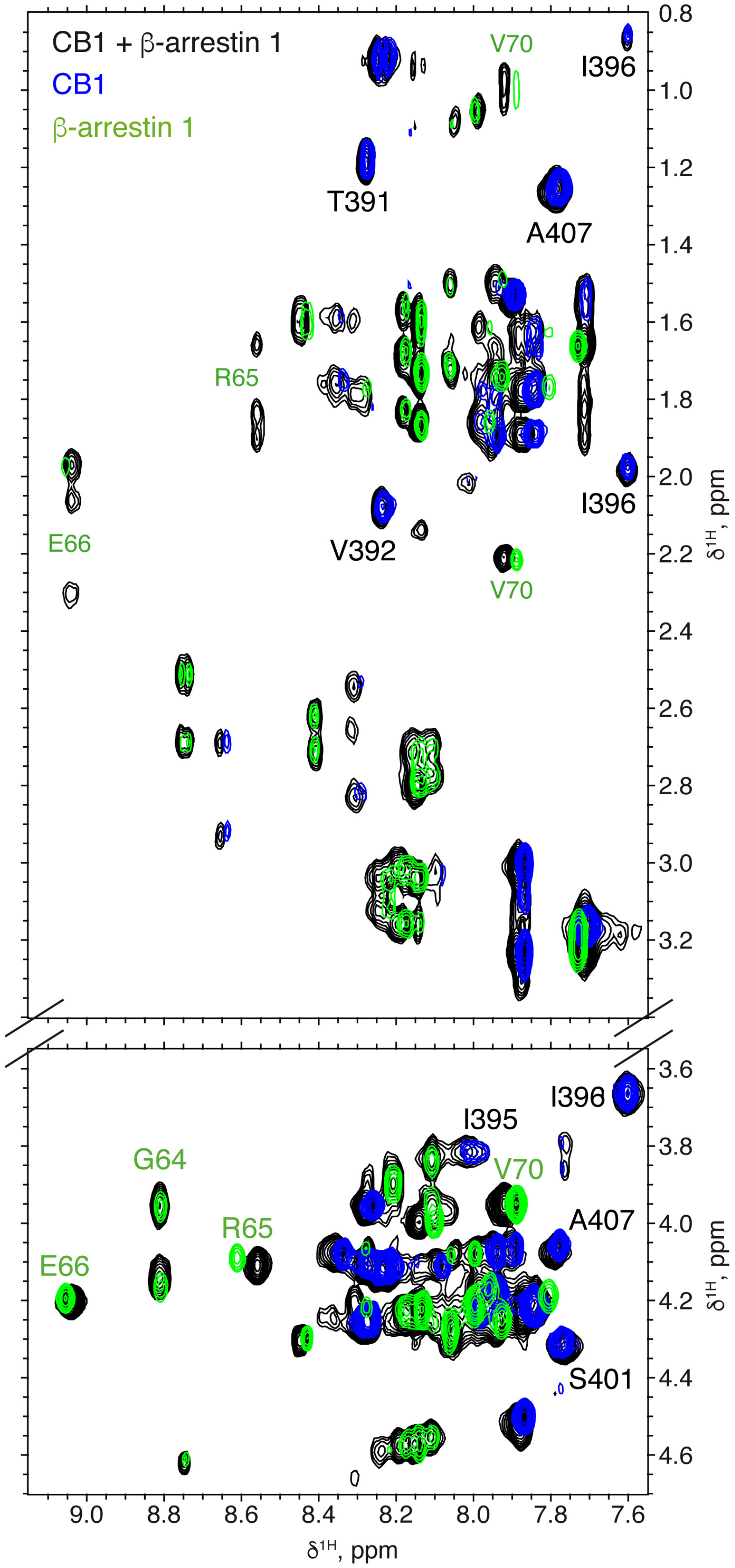

Figure S6. Overlay of selected regions of 2D ^{1}H,^{1}H TOCSY spectra for CB1$^{391\text{-}409}$+ β-arr1$^{63\text{-}76}$ (black contours), CB1$^{391\text{-}409}$ (blue contours) and β-arr1$^{63\text{-}76}$ (green contours) in DPC micelles at 25 ºC. Vertical is split to avoid showing regions without any cross-peak.

Table S1. Design of β–arrestin1 finger loop peptide (β-arr1$^{63-76}$; top): Sequence alignment of arrestins (middle) and taken-into-account parameters (bottom). A the top panel, absolute and relative peptide numberings are shown.

β-arr1$^{63-76}$	63	64	65	66	67	68	69	70	71	72	73	74	75	76
	Y	G	R	E	D	L	D	V	L	G	L	T	F	R
	1	2	3	4	5	6	7	8	9	10	11	12	13	14

Arrestin subtype	Species							Finger loop (FL)																
Arrestin-S	Human	T	C	A	F	R	Y	G	Q	E	D	I	D	V	I	G	L	T	F	R	R	D	L	Y
Arrestin-S	Bovine	T	C	A	F	R	Y	G	Q	E	D	I	D	V	M	G	L	S	F	R	R	D	L	Y
Arrestin-S	Mouse	T	C	A	F	R	Y	G	Q	E	D	I	D	V	M	G	L	T	F	R	R	D	L	Y
β-arrestin 1	Human	T	C	A	F	R	Y	G	R	E	D	L	D	V	L	G	L	T	F	R	K	D	L	F
β-arrestin 1	Bovine	T	C	A	F	R	Y	G	R	E	D	L	D	V	L	G	L	T	F	R	K	D	L	F
β-arrestin 1	Rat	T	C	A	F	R	Y	G	R	E	D	L	D	V	L	G	L	T	F	R	K	D	L	F
β-arrestin 1	Mouse	T	C	A	F	R	Y	G	R	E	D	L	D	V	L	G	L	T	F	R	K	D	L	F
β-arrestin 2	Human	T	C	A	F	R	Y	G	R	E	D	L	D	V	L	G	L	S	F	R	K	D	L	F
β-arrestin 2	Bovine	T	C	A	F	R	Y	G	R	E	D	L	D	V	L	G	L	S	F	R	K	D	L	F
β-arrestin 2	Rat	T	C	A	F	R	Y	G	R	E	D	L	D	V	L	G	L	S	F	R	K	D	L	F
β-arrestin 2	Mouse	T	C	A	F	R	Y	G	R	E	D	L	D	V	L	G	L	S	F	R	K	D	L	F
Arrestin-C	Human	T	C	A	F	R	Y	G	R	D	D	L	E	V	I	G	L	T	F	R	K	D	L	Y
Arrestin-C	Mouse	T	C	A	F	R	Y	G	R	D	D	L	D	V	I	G	L	T	F	R	K	D	L	Y

	β–arr1 FL peptide	Nº aa	MW	pI[a]	Predicted Helicity[b]	Non-polar residues		Charged residues	
						Number	%	+	-
**1**	AFRYGREDLDVLGLTFRK	18	2156.47	8.63	AFRYGREDLDVLGLTFRK	9 (1 A; 2 G;3 L; 2 F; 1 V)	50.1	4	3
**2**	RYGREDLDVLGLTFRK	16	1938.22	8.59	RYGREDLDVLGLTFRK	8 (2 G; 3 L; 2 F; 1 V)	43.7	4	3
**3**	YGREDLDVLGLTFRK	15	1782.03	6.12	YGREDLDVLGLTFRK	7 (2 G; 3 L; 1 F; 1 V)	46.7	3	3
**4**	GREDLDVLGLTFRK	14	1618.85	6.12	GREDLDVLGLTFRK	7 (2 G; 3 L; 1 F; 1 V)	49.9	3	3
**5[c]**	YGREDLDVLGLTFR[c]	14	1653.86	4.56	YGREDLDVLGLTFR	7 (2 G; 3 L; 1 F; 1 V)	49.9	2	3
**6**	YGREDLDVLGLT	12	1350.49	4.03	YGREDLDVLGLT	6 (2 G; 3 L; 1 V)	50.0	1	3
**7**	YGREDLDVLGLTF	13	1497.67	4.03	YGREDLDVLGLTF	7 (2 G; 3 L; 1 F; 1 V)	53.9	1	3

[a] *ProtParam* (predicted values at pH 7)
[b] *Agadir* prediction; conditions: 278 K; pH: 5.5; Ionic strength: 0.1; Nter acetylated; Cter amidated; Probability of helical residues: 1.5–1.3% yellow; 1.2–1.0% cyan.
[c] Selected peptide: β-arr1$^{63-76}$

Table S2. CB1 TMH7-H8 peptide design.

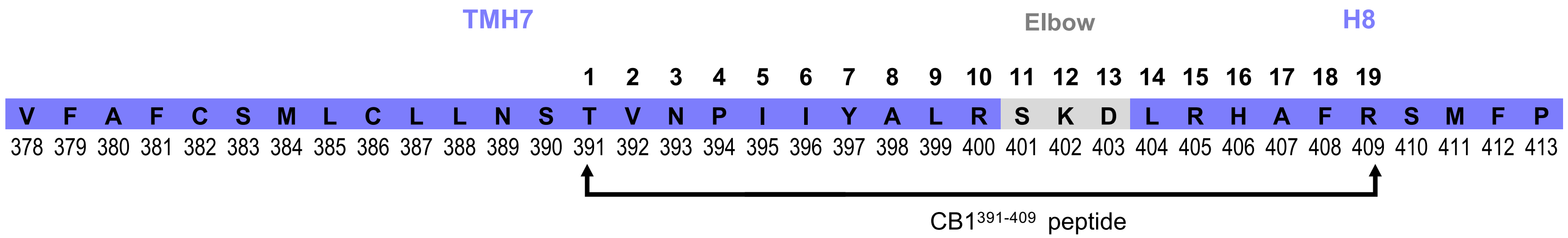

CB1 peptide		Nº aa	MW	pI[a]	Predicted Helicity[b]	Non-polar residues		Charged residues	
						Number	%	+	-
1	LLNSTVNPIIYALRSKDLRHAFR	23	2698.17	10.90	LLNSTVNPIIYALRSKDLRHAFR	10 (2 A;2 I;4 L;1 F;1 V;1P)	43.4	4	1
2	NSTVNPIIYALRSKDLRHAFR	21	2471.85	10.90	NSTVNPIIYALRSKDLRHAFR	9 (2 A;2 I;2 L;1 F;1 V;1 P)	42.9	4	1
3	NSTVNPIIYALRSKDLRHA	19	2168.48	9.99	NSTVNPIIYALRSKDLRHA	8 (2 A; 2 I; 2 L; 1 V; 1 P)	42.1	3	1
4	NSTVNPIIYALRSKDLR	17	1960.26	9.99	NSTVNPIIYALRSKDLR	8 (1 A;2 I;2 L;1 F;1 V;1 P)	41.3	3	1
5[c]	TVNPIIYALRSKDLRHAFR	19	2270.67	10.90	TVNPIIYALRSKDLRHAFR	9 (2 A;2 I;2 L;1 F;1 V;1 P)	47.4	4	1
6	TVNPIIYALRSKDLRHA	17	1967.30	9.98	TVNPIIYALRSKDLRHA	8 (2 A; 2 I; 2 L; 1 V; 1 P)	47.2	3	1
7	VNPIIYALRSKDLRH	15	1795.12	9.99	VNPIIYALRSKDLRH	7 (1 A;2 I;2 L;1 F;1 V;1 P)	46.7	3	1
8	NPIIYALRSKDLR	13	1558.84	9.99	NPIIYALRSKDLR	6 (1 A; 2 I;2 L;1 F; 1 P)	46.2	3	1
9	NPIIYALRSKDLRHAFR	17	2070.43	10.90	NPIIYALRSKDLRHAFR	8 (2 A 2 I;2 L;1 F; 1 P)	47.2	4	1
10	VNPIIYALRSKDLRHAFR	18	2169.56	10.90	VNPIIYALRSKDLRHAFR	9 (2 A 2 I;2 L;1 F;1 V;1 P)	50.1	4	1
11	VNPIIYALRSKDLRHAFRS	19	2256.64	10.90	VNPIIYALRSKDLRHAFRS	9 (2 A 2 I;2 L;1 F;1 V;1 P)	47.4	4	1
12	VNPIIYALRSKDLRHAF	17	2013.37	9.99	VNPIIYALRSKDLRHAF	9 (2 A 2 I;2 L;1 F;1 V;1 P)	53.1	3	1
13	VNPIIYALRSKDLRHA	16	1866.20	9.99	VNPIIYALRSKDLRHA	8 (2 A 2 I; 2 L; 1 V; 1 P)	49.9	3	1

[a] *ProtParam* (predicted values at pH 7)
[b] *Agadir* prediction; conditions: 278 K; pH: 5.5; Ionic strength: 0.1; Nter acetylated; Cter amidated; Probability of helical residues: > 2% yellow; 2–1% cyan; 1-0.8 grey.
[c] Selected peptide: $CB1^{391-409}$

Table S3. ^{1}H and ^{13}C chemical shifts (ppm, from DSS) for β-arr1$^{63\text{-}76}$ in H_2O/D_2O (9:1 ratio by volume) pH 5.5 at 5°C. BMRB ID: 50377

Residue	HN	CαH	$^{13}C_\alpha$	CβH	$^{13}C_\beta$	Others
CH_3CO		1.94	24.4			
Y63	8.42	4.49	58.5	3.06, 2.92	38.8	2,6H 7.14, 7.14; 3,5H 6.83, 6.83
G64	8.68	3.89, 3.89	45.3	-	-	
R65	8.22	4.33	56.2	1.90, 1.76	30.8	γCH_2 1.60, 1.60; δCH_2 3.19, 3.19; εNH 7.52
E66	8.83	4.27	57.1	2.06, 1.93	29.9	γCH_2 2.29, 2.29
D67	8.49	4.57	54.4	2.72, 2.62	40.6	
L68	8.16	4.28	55.5	1.55, 1.67	42.4	γCH 1.61; δCH_3 0.82, 0.90
D69	8.42	4.60	54.5	2.73, 2.68	40.7	
V70	8.05	3.99	63.4	2.15	32.3	γCH_3 0.95, 0.93
L71	8.28	4.29	55.7	1.76, 1.57	42.0	γCH 1.64; δCH_3 0.86, 0.91
G72	8.30	3.93, 3.93	45.8	-	-	
L73	8.22	4.34	55.7	1.71, 1.57	42.4	γCH 1.62; δCH_3 0.86, 0.93
T74	8.20	4.26	62.4	4.14	69.9	γCH_3 1.14
F75	8.33	4.57	58.3	3.09, 3.09	39.5	2,6H 7.25, 7.25; 3,5H 7.35, 7.35; 4H 7.27
R76	8.30	4.21	55.8	1.81, 1.64	30.8	γCH_2 1.56, 1.56; δCH_2 3.15, 3.15; εNH 7.23
$CONH_2$	6.84, 7.13					

Table S4. ^{1}H and ^{13}C chemical shifts (ppm, from DSS) for $CB1^{391\text{-}409}$ in H_2O/D_2O (9:1 ratio by volume) pH 5.5 at 5°C. BMRB ID: 50374

Residue	HN	$C_\alpha H$	$^{13}C_\alpha$	$C_\beta H$	$^{13}C_\beta$	Others
CH_3CO		2.06	24.4			
T391	8.35	4.27	62.3	4.11	69.9	γCH_3 1.18
V392	8.40	4.10	62.1	2.02	32.9	γCH_3 0.93, 0.89
N393	8.72	-	-	2.85, 2.72	38.7	γNH_2 7.05, 7.79
P394	-	4.39	63.5	2.29, 1.91	32.3	γCH_2 2.02, 2.02; δCH_2 3.87, 3.76
I395	8.23	3.97	61.8	1.76	38.2	γCH_2 1.50, 1.16; γCH_3 0.69; δCH_3 0.86
I396	8.04	4.05	61.1	1.81	38.2	γCH_2 1.39, 1.17; γCH_3 0.83; δCH_3 0.81
Y397	8.37	4.43	58.8	3.03, 2.90	38.8	2,6H 7.11, 7.11; 3,5H 6.80, 6.80
A398	8.25	4.23	52.9	1.39	19.1	
L399	8.13	4.28	55.5	1.60, 1.68	42.2	γCH 1.66; δCH_3 0.89, 0.94
R400	8.32	4.32	56.4	1.87, 1.78	30.6	γCH_2 1.65; δCH_2 3.16; εNH 7.25
S401	8.32	4.34	58.8	3.89, 3.82	63.7	
K402	8.41	4.25	56.9	1.84, 1.78	32.8	γCH_2 1.46, 1.40; δCH_2 1.67; εCH_2 2.98; εNH_3^+ 7.61
D403	8.33	4.60	54.1	2.80, 2.64	40.7	
L404	8.36	4.28	55.5	1.58, 1.66	42.0	γCH 1.64; δCH_3 0.85, 0.93
R405	8.29	4.20	56.7	1.76, 1.76	30.4	γCH_2 1.54, 1.61; δCH_2 3.17, 3.17; εNH 7.40
H406	8.41	4.65	55.2	3.25, 3.12	29.0	2H 8.61; 4H 7.27
A407	8.38	4.28	52.5	1.33	19.2	
F408	8.51	4.58	58.1	3.12, 3.06	39.6	2,6H 7.28, 7.28; 3,5H 7.36, 7.36; 4H 7.30
R409	8.38	4.21	55.7	1.64, 1.80	30.8	γCH_2 1.56, 1.56; δCH_2 3.15, 3.15; εNH 7.22
$CONH_2$	6.90, 7.13					

Table S5. ^1H and ^{13}C chemical shifts (ppm, from DSS) for β-arr1$^{63-76}$ in 70% H_2O/D_2O (9:1 ratio by volume)/ 30% TFE pH 5.5 at 25°C. BMRB ID: 50376

Residue	HN	CαH	$^{13}C_\alpha$	CβH	$^{13}C_\beta$	Others
CH_3CO		1.98	24.2			
Y63	8.05	4.54	58.4	3.09, 2.93	39.0	2,6H 7.16, 7.16; 3,5H 6.86, 6.86
G64	8.50	3.94, 4.01	45.2	-	-	
R65	8.01	4.39	56.3	1.98, 1.83	30.9	γCH_2 1.68, 1.68; δCH_2 3.23, 3.23; εNH 7.81
E66	8.91	4.29	58.0	2.13, 2.04	29.4	γCH_2 2.39, 2.39
D67	8.34	4.53	55.1	2.74	40.2	
L68	7.84	4.26	56.3	1.75, 1.60	42.2	γCH 1.68; δCH_3 0.86, 0.93
D69	8.18	4.63	55.5	2.80, 2.80	40.0	
V70	7.89	3.85	65.3	2.19	32.0	γCH_3 1.06, 0.98
L71	8.05	4.17	57.4	1.83, 1.63	41.7	γCH 1.73, 1.73; δCH_3 0.89, 0.93
G72	8.15	3.90, 3.94	46.7	-	-	
L73	8.04	4.23	57.0	1.86, 1.62	42.2	γCH 1.71; δCH_3 0.90, 0.93
T74	8.00	4.10	64.3	4.18	69.6	γCH_3 1.09
F75	7.96	4.61	58.8	3.25, 3.12	39.0	2,6H 7.30, 7.30; 3,5H 7.33, 7.33; 4H 7.27
R76	7.88	4.28	56.3	1.95, 1.83	30.7	γCH_2 1.67, 1.74; δCH_2 3.22, 3.22; εNH 7.23
$CONH_2$	6.94, 7.00					

Table S6. ^{1}H and ^{13}C chemical shifts (ppm, from DSS) for $CB1^{391-409}$ in 70% H_2O/D_2O (9:1 ratio by volume)/ 30% TFE pH 5.5 at 25°C. BMRB ID: 50373

Residue	HN	$C_{\alpha}H$	$^{13}C_{\alpha}$	$C_{\beta}H$	$^{13}C_{\beta}$	Others
CH_3CO		2.10	24.2			
T391	7.92	4.37	61.8	4.19	70.0	γCH_3 1.23
V392	7.91	4.15	61.8	2.05	33.3	γCH_3 0.94, 0.89
N393	7.87	4.92	51.6	2.89	39.0	γNH_2 6.43, 7.66
P394	-	4.35	65.2	2.01, 2.43	32.2	γCH_2 2.08, 2.08; δCH_2 3.82, 3.97
I395	7.91	3.91	64.1	2.00	37.5	γCH_2 1.60, 1.27; γCH_3 0.91; δCH_3 0.91
I396	7.50	3.77	63.9	1.92	37.3	γCH_2 1.55, 1.30; γCH_3 0.91; δCH_3 0.88
Y397	7.89	4.09	61.5	3.04, 3.09	38.2	2,6H 7.07, 7.07; 3,5H 6.81, 6.81
A398	8.09	4.04	55.3	1.61	17.8	
L399	8.33	4.12	57.8	1.54, 1.94	42.1	γCH 1.93; δCH_3 0.87, 0.87
R400	8.33	4.21	57.9	1.83, 1.91	30.0	γCH_2 1.70, 1.83; δCH_2 3.09, 3.15
S401	7.92	4.23	60.4	3.77, 3.93	63.4	
K402	7.74	4.21	57.8	1.96, 1.96	32.3	γCH_2 1.53, 1.50; δCH_2 1.71, 1.71; εCH_2 3.01, 3.01
D403	8.00	4.66	55.1	2.70, 2.87	41.0	
L404	8.08	4.24	56.5	1.67,1.74	41.9	γCH 1.74; δCH_3 0.90, 0.95
R405	8.09	4.09	58.4	1.82, 1.85	30.0	γCH_2 1.64, 1.68; δCH_2 3.16, 3.23
H406	7.88	4.52	57.5	3.20, 3.11	30.5	2H 7.75; 4H 7.04
A407	7.85	4.19	53.4	1.32	18.5	
F408	7.93	4.60	58.1	3.09, 3.24	39.1	2,6H 7.30, 7.30; 3,5H 7.34, 7.34; 4H 7.28
R409	7.84	4.29	56.0	1.79, 1.91	30.7	γCH_2 1.66, 1.66; δCH_2 3.21, 3.21
$CONH_2$	6.91, 7.02					

Table S7. ^{1}H and ^{13}C chemical shifts (ppm, from DSS) for β-arr1$^{63\text{-}76}$ in a H_2O/D_2O (9:1 ratio by volume) solution containing 30 mM [D_{38}]-DPC at 25°C. BMRB ID: 50375

Residue	HN	CαH	^{13}Cα	CβH	^{13}Cβ	Others
CH_3CO		1.93	24.7			
Y63	8.14	4.58	58.4*	2.79, 3.04	39.8	2,6H 7.09, 7.09; 3,5H 6.79, 6.79
G64	8.81	3.96, 4.17	45.1			
R65	8.61	4.10	58.1	1.85, 1.91	30.5	γCH_2 1.67, 1.67; δCH_2 3.18, 3.23; εNH 7.23
E66	9.06	4.20	58.1	1.98, 2.06	29.2	γCH_2 2.31, 2.31
D67	8.10	4.55	55.6*	2.71, 2.78	41.0	
L68	7.80	4.20	56.0	1.64, 1.77	42.1	γCH 1.72; δCH_3 0.84, 0.92
D69	8.14	4.55	55.2*	2.72, 2.72	40.5	
V70	7.89	3.96	64.2	2.22	32.1	γCH_3 1.04, 0.98
L71	7.96	4.18	56.4	1.62, 1.86	41.9	γCH 1.75; δCH_3 0.88, 0.93
G72	8.11	3.84, 3.95	45.1			
L73	7.93	4.25	55.8	1.50, 1.75	42.5	γCH 1.66; δCH_3 0.87, 0.91
T74	8.00	4.22	63.1	4.09	69.8	γCH_3 1.06
F75	8.17	4.57	58.2*	3.02, 3.16	39.7	2,6H 7.27, 7.27; 3,5H 7.27, 7.27; 4H 7.18
R76	8.13	4.22	56.1	174,1.87	31.0	γCH_2 1.59, 1.64; δCH_2 3.17, 3.17; εNH 7.42
$CONH_2$	7.13, 7.24					

*Assigned from the spectra in pure D_2O.

Table S8. ^{1}H and ^{13}C chemical shifts (ppm, from DSS) for $CB1^{391-409}$ in a H_2O/D_2O (9:1 ratio by volume) solution containing 30 mM [D_{38}]-DPC at 25°C. BMRB ID: 50372

Residue	HN	$C_\alpha H$	$^{13}C_\alpha$	$C_\beta H$	$^{13}C_\beta$	Others
CH_3CO		2.07	24.7			
T391	8.27	4.26	62.8	4.11	69.9	γCH_3 1.19
V392	8.23	4.12	62.2	2.08	33.0	γCH_3 0.90, 0.94
N393	8.64	-	-	2.93, 2.70	38.2	γNH_2 6.73, 8.09
P394	-	4.36	65.4*	1.88, 2.42	32.3	γCH_2 2.06, 2.06; δCH_2 3.74, 3.85
I395	8.00	3.82	64.3	2.02	37.6	γCH_2 1.66, 1.19; γCH_3 0.87; δCH_3 0.91
I396	7.60	3.67	64.1	1.98	37.2	γCH_2 1.54, 1.20; γCH_3 0.86; δCH_3 0.85
Y397	8.08	4.12	60.9	3.02, 3.07	38.1	2,6H 7.08, 7.08; 3,5H 6.80, 6.80
A398	7.89	4.08	54.7	1.54	18.7	
L399	7.94	4.08	57.1	1.52, 1.91	42.2	γCH 1.92; δCH_3 0.85, 0.87
R400	7.94	4.19	57.5*	1.80, 1.91	30.8	γCH_2 1.66, 1.80; δCH_2 3.09, 3.09; εNH 7.45
S401	7.77	4.31	59.3*	3.79, 3.87	63.9	
K402	7.98	4.23	56.2*	1.78, 1.86	32.8	γCH_2 1.44, 1.47; δCH_2 1.66, 1.66; εCH_2 2.92, 2.92
D403	8.30	4.68*	53.9*	2.54, 2.83	40.6	
L404	8.34	4.09	57.1	1.60, 1.76	42.1	γCH 1.78; δCH_3 0.87, 0.96
R405	8.26	3.96	58.9	1.77, 1.81	29.9	γCH_2 1.53, 1.58; δCH_2 3.18, 3.18; εNH 7.71
H406	7.87	4.60*	56.6*	3.09, 3.28	29.5	2H 8.21; 4H 7.16
A407	7.78	4.07	54.0	1.26	18.8	
F408	7.87	4.50	58.2*	3.01, 3.23	39.5	2,6H 7.28, 7.28; 3,5H 7.22, 7.22; 4H 7.12
R409	7.84	4.22	56.1	1.79, 1.89	30.9	γCH_2 1.62, 1.66; δCH_2 3.16, 3.16; εNH 7.45
$CONH_2$	7.19, 7.39					

*Assigned from the spectra in pure D_2O.

Table S9. ^{1}H and ^{13}C chemical shifts (ppm, from DSS) for the mixture of β-arr1$^{63-76}$ and CB1$^{391-409}$ peptides in H_2O/D_2O (9:1 ratio by volume) pH 5.5 at 5°C. BMRB ID: 50382

Residue	HN	CαH	$^{13}C_{\alpha}$	CβH	$^{13}C_{\beta}$	Others
CH_3CO		1.95	24.4			
Y63	8.40	4.48	58.5	3.05, 2.91	38.8	2,6H 7.13, 7.13; 3,5H 6.84, 6.84
G64	8.69	3.89	45.3	-	-	
R65	8.22	4.33	56.2	1.90, 1.76	30.8	γCH_2 1.60, 1.60; δCH_2 3.20, 3.20; εNH 7.53
E66	8.85	4.27	57.1	2.06, 1.93	30.0	γCH_2 2.28, 2.28
D67	8.48	4.57	54.4	2.72, 2.62	40.8	
L68	8.15	4.28	55.5	1.55, 1.67	42.4	γCH 1.61; δCH_3 0.82, 0.90
D69	8.42	4.60	54.5	2.73, 2.67	40.8	
V70	8.05	3.99	63.4	2.16	32.3	γCH_3 0.95, 0.93
L71	8.28	4.29	55.7	1.76, 1.57	42.0	γCH 1.64; δCH_3 0.85, 0.91
G72	8.30	3.93	45.8	-	-	
L73	8.22	4.34	55.7	1.70, 1.57	42.4	γCH 1.62; δCH_3 0.87, 0.93
T74	8.20	4.26	62.4	4.14	69.9	γCH_3 1.15
F75	8.33	4.57	58.3	3.21, 3.09	39.5	2,6H 7.26, 7.26; 3,5H 7.35, 7.35; 4H 7.30
R76	8.31	4.20	55.8	1.81, 1.64	30.8	γCH_2 1.56, 1.56; δCH_2 3.15, 3.15; εNH 7.23
$CONH_2$	6.85, 7.14					
CH_3CO		2.07	24.4			
T391	8.35	4.27	62.3	4.11	69.9	γCH_3 1.18
V392	8.41	4.11	62.1	2.02	32.9	γCH_3 0.93, 0.90
N393	8.73	-	-	2.85, 2.72	38.7	γNH_2 7.06, 7.79
P394	-	4.39	63.5	2.29, 1.91	32.3	γCH_2 2.02, 2.02; δCH_2 3.87, 3.77
I395	8.23	3.97	61.8	1.76	38.3	γCH_2 1.50, 1.16; γCH_3 0.69; δCH_3 0.86
I396	8.05	4.05	61.1	1.81	38.3	γCH_2 1.39, 1.17; γCH_3 0.82; δCH_3 0.81
Y397	8.37	4.42	58.8	3.04, 2.90	38.8	2,6H 7.23, 7.23; 3,5H 6.80, 6.80
A398	8.17	4.23	52.9	1.39	19.1	
L399	8.13	4.28	55.5	1.60, 1.68	42.2	γCH 1.66; δCH_3 0.89, 0.94
R400	8.32	4.32	56.3	1.87, 1.78	30.6	γCH_2 1.65, 1.65; δCH_2 3.15, 3.15; εNH 7.25
S401	8.33	4.34	58.8	3.90, 3.82	63.7	
K402	8.43	4.26	57.0	1.85, 1.78	32.8	γCH_2 1.45, 1.40; δCH_2 1.67, 1.67; εCH_2 2.97, 2.97
D403	8.32	4.59	54.3	2.78, 2.61	41.1	
L404	8.36	4.28	55.5	1.58, 1.66	42.0	γCH 1.64; δCH_3 0.85, 0.93
R405	8.30	4.19	56.7	1.76	30.4	γCH_2 1.56, 1.61; δCH_2 3.16, 3.16; εNH 7.43
H406	8.33	4.62	55.5	3.20, 3.08	29.6	2H 8.35; 4H 7.18
A407	8.32	4.27	52.6	1.32	19.2	
F408	8.48	4.57	58.1	3.13, 3.07	39.6	2,6H 7.28, 7.28; 3,5H 7.36, 7.36; 4H 7.30
R409	8.37	4.21	55.7	1.64, 1.80	30.8	γCH_2 1.56, 1.56; δCH_2 3.15, 3.15; εNH 7.22
$CONH_2$	6.91, 7.14					

Table S10. ^{1}H and ^{13}C chemical shifts (ppm, from DSS) for the mixture of β-arr1$^{63-76}$ and CB1$^{391-409}$ peptides in 70% H_2O/D_2O (9:1 ratio by volume)/ 30% TFE pH 5.5 at 25°C. BMRB ID: 50383

Residue	HN	$C_\alpha H$	$^{13}C_\alpha$	$C_\beta H$	$^{13}C_\beta$	Others
CH_3CO		1.98	24.2			
Y63	8.05	4.55	58.4	3.09, 2.93	39.0	2,6H 7.16, 7.16; 3,5H 6.87, 6.87
G64	8.54	3.97	45.2	-	-	
R65	8.02	4.38	56.4	1.98, 1.83	30.9	γCH_2 1.68, 1.68; δCH_2 3.23, 3.23; εNH 7.81
E66	8.98	4.28	58.2	2.12, 2.02	29.6	γCH_2 2.39, 2.39
D67	8.34	4.54	55.2	2.73	40.4	
L68	7.84	4.26	56.3	1.75, 1.60	42.2	γCH 1.68; δCH_3 0.86, 0.93
D69	8.18	4.64	55.4	2.79	40.3	
V70	7.89	3.85	65.3	2.19	32.0	γCH_3 1.06, 0.98
L71	8.05	4.17	57.4	1.83, 1.63	41.7	γCH 1.73; δCH_3 0.90, 0.93
G72	8.15	3.94	46.8	-	-	
L73	8.05	4.23	57.0	1.86, 1.62	42.2	γCH 1.77; δCH_3 0.90, 0.93
T74	8.00	4.10	64.3	4.18	69.6	γCH_3 1.09
F75	7.96	4.61	58.8	3.25, 3.12	39.0	2,6H 7.30, 7.30; 3,5H 7.33, 7.33; 4H 7.28
R76	7.88	4.29	56.3	1.95, 1.83	30.7	γCH_2 1.68, 1.74; δCH_2 3.22, 3.22; εNH 7.23
$CONH_2$	6.90, 7.03					
CH_3CO		2.10	24.2			
T391	7.89	4.38	61.8	4.19	69.9	γCH_3 1.23
V392	7.89	4.15	61.9	2.06	33.3	γCH_3 0.89, 0.94
N393	7.85	4.93	51.6	2.89, 2.89	39.0	γNH_2 6.41, 7.64
P394	-	4.36	65.3	2.01, 2.43	32.2	γCH_2 2.08, 2.08; δCH_2 3.82, 3.97
I395	7.91	3.91	64.1	2.00	37.5	γCH_2 1.60, 1.28; γCH_3 0.91; δCH_3 0.91
I396	7.50	3.77	64.0	1.92	37.3	γCH_2 1.55, 1.30; γCH_3 0.91; δCH_3 0.88
Y397	7.89	4.09	61.6	3.04, 3.10	38.2	2,6H 7.07, 7.07; 3,5H 6.81, 6.81
A398	8.09	4.04	55.4	1.61	17.7	
L399	8.35	4.12	57.8	1.54, 1.95	42.1	γCH 1.92; δCH_3 0.87, 0.87
R400	8.37	4.21	58.0	1.84, 1.91	30.0	γCH_2 1.70, 1.82; δCH_2 3.09, 3.15
S401	7.94	4.22	60.5	3.77, 3.93	63.4	
K402	7.74	4.21	57.9	1.96, 1.96	32.2	γCH_2 1.50, 1.54; δCH_2 1.72, 1.72; εCH_2 3.02, 3.02
D403	7.99	4.69	54.8	2.72, 2.86	41.0	
L404	8.08	4.24	56.5	1.67, 1.74	41.9	γCH 1.74; δCH_3 0.90, 0.95
R405	8.09	4.09	58.3	1.82, 1.85	30.0	γCH_2 1.64, 1.69; δCH_2 3.16, 3.23
H406	7.91	4.54	57.3	3.22, 3.13	30.0	2H 7.96; 4H 7.12
A407	7.89	4.20	53.5	1.33	18.5	
F408	7.95	4.60	58.1	3.10, 3.24	39.1	2,6H 7.30, 7.30; 3,5H 7.34, 7.34; 4H 7.28
R409	7.84	4.29	56.0	1.79, 1.92	30.8	γCH_2 1.66, 1.66; δCH_2 3.21, 3.21
$CONH_2$	6.91, 7.02					

Table S11. ^{1}H and ^{13}C chemical shifts (ppm, from DSS) for the mixture of β-arr1$^{63-76}$ and CB1$^{391-409}$ peptides in a H_2O/D_2O (9:1 ratio by volume) solution containing 30 mM [D_{38}]-DPC at 25°C. BMRB ID: 50384

Residue	HN	$C_\alpha H$	$^{13}C_\alpha$	$C_\beta H$	$^{13}C_\beta$	Others
CH_3CO		1.93	24.7			
Y63	8.15	4.58	58.4	2.80, 3.04	39.8	2,6H 7.09, 7.09; 3,5H 6.80, 6.80
G64	8.81	4.05	45.2	-	-	
R65	8.56	4.11	57.9	1.84, 1.91	30.6	γCH_2 1.67, 1.67; δCH_2 3.24, 3.24; εNH 7.71
E66	9.04	4.21	58.1	1.97, 2.07	29.3	γCH_2 2.31, 2.31
D67	8.11	4.55	55.5	2.71, 2.76	41.1	
L68	7.84	4.21	55.7	1.64, 1.77	42.1	γCH 1.63; δCH_3 0.84, 0.91
D69	8.15	4.57	55.4	2.72	40.6	
V70	7.92	3.96	64.2	2.21	32.1	γCH_3 1.03, 0.97
L71	7.98	4.18	56.3	1.61, 1.86	41.9	γCH nd; δCH_3 0.86, 0.92
G72	8.11	3.90	45.1	-	-	
L73	7.94	4.26	55.6	1.50, 1.74	42.5	γCH 1.74; δCH_3 0.87, 0.93
T74	7.99	4.21	63.0	4.09	69.9	γCH_3 1.06
F75	8.19	4.59	58.1	3.02, 3.16	39.7	2,6H 7.27, 7.27; 3,5H 7.27, 7.27; 4H 7.18
R76	8.14	4.24	56.0	1.73, 1.87	31.0	γCH_2 1.58, 1.63; δCH_2 3.17, 3.17; εNH 7.41
$CONH_2$	7.13, 7.26					
CH_3CO	2.08	24.7				
T391	8.28	4.26	62.8	4.11	69.9	γCH_3 1.19
V392	8.24	4.13	62.2	2.08	33.0	γCH_3 0.90, 0.94
N393	8.66	-	-	2.94, 2.70	-	γNH_2 6.73, 8.10
P394	-	4.36	65.4*	1.88, 2.42	32.3	γCH_2 2.06, 2.06; δCH_2 3.74, 3.86
I395	8.02	3.82	64.4	2.02	37.6	γCH_2 1.67, 1.19; γCH_3 0.87; δCH_3 0.91
I396	7.60	3.67	64.1	1.99	37.1	γCH_2 1.56, 1.22; γCH_3 0.87; δCH_3 0.85
Y397	8.10	4.11	61.0	3.03, 3.07	38.1	2,6H 7.08, 7.08; 3,5H 6.80, 6.80
A398	7.90	4.08	54.8	1.54	18.6	
L399	7.95	4.08	57.2	1.54, 1.91	42.2	γCH 1.92; δCH_3 0.85, 0.87
R400	7.95	4.18	57.7*	1.81, 1.91	-	γCH_2 1.67, 1.67; δCH_2 3.09, 3.09; εNH 7.46
S401	7.76	4.33	59.3*	3.79, 3.87	63.8	
K402	7.98	4.25	56.1*	1.78, 1.86	32.8	γCH_2 1.44, 1.47; δCH_2 1.66, 1.66; εCH_2 2.92, 2.92
D403	8.31	4.67	53.8*	2.55, 2.84	40.5	
L404	8.35	4.08	57.2	1.60, 1.77	42.1	γCH 1.76; δCH_3 0.87, 0.96
R405	8.28	3.97	58.9	1.77, 1.80	29.9	γCH_2 1.52, 1.57; δCH_2 3.18, 3.18; εNH 7.71
H406	7.88	4.60	56.6*	3.11, 3.31	29.2	2H 8.33; 4H 7.21
A407	7.79	4.08	54.1	1.26	18.9	
F408	7.88	4.51	58.2*	3.02, 3.23	39.5	2,6H 7.28, 7.28; 3,5H 7.23, 7.23; 4H 7.12
R409	7.87	4.23	56.0*	1.79, 1.90	30.9	γCH_2 1.62, 1.66; δCH_2 3.16; εNH 7.45
$CONH_2$	7.20, 7.40					

*Assigned from the spectra in pure D_2O.

Table S12. Summary of structural statistics parameters for the ensemble of the 20 lowest target function conformers calculated for peptides CB1$^{391-409}$ and β-arr1$^{63-76}$ in 30% TFE and in DPC micelles.

	**CB1$^{391-409}$**		**β-arr1$^{63-76}$**	
	**TFE**	**DPC**	**TFE**	**DPC**
**Number of distance restraints**				
Intraresidue & sequential (i – j ≤ 1)	63	157	92	111
Medium range (1 < |i – j| < 5)	15	36	7	18
Long range (|i – j| ≥ 5)	0	0	0	0
Total number	78	193	99	129
Averaged total number per residue	4.1	10.2	7.1	9.2
**Number of dihedral angle constraints**				
Number of restricted ϕ angles	16	14	11	11
Number of restricted ψ angles	15	14	10	11
Total number	31	28	21	22
**Pairwise RMSD (Å)**				
**Residues 392-407**				
Backbone atoms	1.3±0.5	2.1±1.0	---	---
All heavy atoms	2.4±0.7	3.2±1.2	---	---
**Residues P394-K402 (Helix 1)**				
Backbone atoms	0.4±0.1	0.2±0.1	---	---
All heavy atoms	1.2±0.2	1.0±0.2	---	---
**Residues L404-A407 (Helix 2)**				
Backbone atoms	0.2±0.1	0.1±0.1	---	---
All heavy atoms	1.5±0.4	1.3±0.3	---	---
**Residues E66-F75 (R65-F75)**				
Backbone atoms	---	---	0.4±0.1	0.5±0.2 (0.6±0.2)
All heavy atoms	---	---	1.2±0.2	1.3±0.2 (1.6±0.3)
**Ramachandran plot (%)**				
Most favoured regions	91.6	89.1	97	100
Additionally allowed regions	8.4	10.3	3	0
Generously allowed regions	0	0.3	0	0
Disallowed regions	0	0.3	0	0

Table S13. CB1$^{391\text{-}409}$ and β-arr1$^{63\text{-}76}$ residues whose chemical shifts are affected upon interaction.

Residue	Experimental conditions			Residue	Experimental conditions		
**CB1$^{391\text{-}409}$**	H_2O	TFE	DPC	**β-arr1$^{63\text{-}76}$**	H_2O	TFE	DPC
R400	✓	✓	✓	R65	-	-	✓
S401	-	✓	-	E66	-	✓	✓
K402	✓	-	✓	L68	-	✓	✓
D403	✓	✓	-	D69	-	✓	✓
R405	✓	-	-	L73	-	-	✓
H406	✓	✓	-				
A407	✓	✓	-				
F408	-	-	✓				

Table S14. Sequence alignment of GPCRs reported in complex with arrestins compared to CB1 at the studied TMH7-H8 region (Rho: rhodopsin receptor; β1-AR: β1-adrenergic receptor, M2: muscarinic receptor 2; NTS1: neurotensin 1 receptor). Ballesteros-Weinstein numbering is detailed at the top of the table.

	7<br>4<br>7	7<br>4<br>8	7<br>4<br>9	7<br>5<br>0	7<br>5<br>1	7<br>5<br>2	7<br>5<br>3	7<br>5<br>4	7<br>5<br>5	7<br>5<br>6	8<br>4<br>7	8<br>4<br>8	8<br>4<br>9	8<br>5<br>0	8<br>5<br>1	8<br>5<br>2	8<br>5<br>3	8<br>5<br>4	8<br>5<br>5
**CB1**	T	V	N	P	I	I	Y	A	L	R	S	K	D	L	R	H	A	F	R
**Rho**	I	Y	N	P	V	I	Y	I	M	M	N	K	Q	F	R	N	C	M	L
**β1-AR**	A	F	N	P	I	I	Y	C	R	-	S	P	D	F	R	K	A	F	Q
**M2**	T	I	N	P	A	C	Y	A	L	C	N	A	T	F	K	K	T	F	K
**NTS1**	T	I	N	P	I	L	Y	N	L	V	S	A	N	F	R	H	I	F	L
